# The KxGxYR and DxE motifs in the C-tail of the Middle East respiratory syndrome coronavirus membrane protein are crucial for infectious virus assembly

**DOI:** 10.1007/s00018-023-05008-y

**Published:** 2023-11-09

**Authors:** Lowiese Desmarets, Adeline Danneels, Julien Burlaud-Gaillard, Emmanuelle Blanchard, Jean Dubuisson, Sandrine Belouzard

**Affiliations:** 1 Université de Lille, CNRS, Inserm, CHU Lille, Institut Pasteur de Lille, U1019-UMR 9017-CIIL-Center for Infection and Immunity of Lille, 59000 Lille, France; 2 INSERM U1259 MAVIVH, Université de Tours and CHRU de Tours, Tours, France; 3 Plate-Forme IBiSA de Microscopie Electronique, Université de Tours and CHRU de Tours, Tours, France

**Keywords:** Coronavirus, Viral assembly, Membrane protein, Intracellular trafficking, Endoplasmic reticulum exit, Trans-Golgi network retention

## Abstract

The coronavirus’ (CoV) membrane (M) protein is the driving force during assembly, but this process remains poorly characterized. Previously, we described two motifs in the C-tail of the Middle East respiratory syndrome CoV (MERS-CoV) M protein involved in its endoplasmic reticulum (ER) exit (_211_DxE_213_) and trans-Golgi network (TGN) retention (_199_KxGxYR_204_). Here, their function in virus assembly was investigated by two different virus-like particle (VLP) assays and by mutating both motifs in an infectious MERS-CoV cDNA clone. It was shown that the _199_KxGxYR_204_ motif was essential for VLP and infectious virus assembly. Moreover, the mislocalization of the M protein induced by mutation of this motif prevented M–E interaction. Hampering the ER export of M by mutating its _211_DxE_213_ motif still allowed the formation of nucleocapsid-empty VLPs, but prevented the formation of fully assembled VLPs and infectious particles. Taken together, these data show that the MERS-CoV assembly process highly depends on the correct intracellular trafficking of its M protein, and hence that not only specific protein–protein interacting motifs but also correct subcellular localization of the M protein in infected cells is essential for virus formation and should be taken into consideration when studying the assembly process.

**Supplementary Information:**

The online version contains supplementary material available at 10.1007/s00018-023-05008-y.

## Introduction

Discovered in the mid twentieth century, CoVs have been associated with high morbidity and mortality in animal species [1, 2]. Before 2002, only human CoVs causing mild upper respiratory tract infections were known, but this changed with the outbreak of the highly pathogenic severe acute respiratory syndrome CoV (SARS-CoV). This outbreak highlighted the intrinsic capacity of CoVs to cross species barriers, and the fear for those zoonotic CoVs further increased with the emergence of the pathogenic Middle East respiratory syndrome coronavirus (MERS-CoV) in 2012, and SARS-CoV-2 in 2019.

Similar to other CoVs, MERS-CoV possesses a very large positive-sense RNA genome of around 30.1 kb that is associated with nucleocapsid (N) proteins to form the helical ribonucleocapsid. This ribonucleocapsid core is surrounded by a lipid envelope in which three structural proteins are embedded, the spike (S), the membrane (M) and the envelope (E) protein. The spike protein (S) is responsible for entry of the virus in its target cell by mediating receptor binding and fusion of the viral membrane with a host cell membrane [3, 4]. The most abundantly present M protein is involved in the viral assembly process and immune evasion [5, 6]. The small E protein has multiple functions during the viral life cycle, particularly in assembly and egress, and it functions as a viroporin [6–8]. The E protein is typically found in only very low numbers in the viral envelope [6, 9, 10]. The primary role of the N protein is essentially structural and consists of protecting the viral genome and ensuring its incorporation into the ribonucleocapsids of the particles. However, N proteins also possess important non-structural functions by interacting with numerous host cells proteins, thereby modulating various cellular processes [11].

CoV assembly and subsequent release is a key determinant of virus spread within or between individuals, and hence might be an attractive target for therapeutic intervention. All virion-associated components are acquired during assembly at the membranes of the endoplasmic reticulum-to-Golgi intermediate compartment (ERGIC) [12, 13]. This assembly process is tightly regulated by complex protein–protein interactions to ensure that all required components of the virus particle are gathered in the ERGIC and are incorporated in the virion, after which they are released by (lysosomal) exocytosis [13–16]. The M protein seems to be the driving force in the CoV assembly process [17, 18]. The MERS-CoV M protein contains 219 amino acids and is composed of a short, N-terminal exodomain that contains 1 N-glycosylation site (N3), three transmembrane helices, and a large C-terminal endodomain that makes up half of the protein. When expressed individually, coronaviral M proteins are retained in the Golgi complex [19–21], although the exact Golgi region and the M domains involved in this retention differ among CoVs [18, 22–26]. The MERS-CoV M protein is typically retained in the trans-Golgi network (TGN) [27, 28], and we previously identified two motifs in the C-tail that are important for the trafficking of the single-expressed MERS-CoV M protein. The _211_DxE_213_ motif is required to export the M protein from the endoplasmic reticulum (ER) upon translation, whereas the _199_KxGxYR_204_ motif subsequently retains the M protein in the TGN [27].

Although M proteins are the driving force during assembly, they cannot act on their own, and other viral proteins, notably the E and/or N proteins, are additionally required for the formation of virus-like particles (VLPs) [6, 7, 29–40]. For most CoVs, the S protein is not involved in VLP formation but seems to be incorporated into virions by interacting with the M protein [6, 41, 42]. So far, it remains largely elusive how the complex assembly process is orchestrated [43]. The aim of the present study was to optimize a reliable VLP assay in mammalian cells as a functional test for the MERS-CoV assembly process, and to assess if the _199_KxGxYR_204_- and _211_DxE_213_-mediated intracellular trafficking/localization of the M protein was necessary for virus assembly.

## Materials and methods

### Plasmid construction for M protein expression

The coding sequence of the MERS-CoV M protein was cloned between the BamHI and EcoRI restriction sites of a pCDNA3.1(+) vector, with either a C-terminal or N-terminal V5-tag, as described before [27]. The M_N3Q_ glycosylation site mutant was generated by site-directed mutagenesis PCR, using Q5® High-Fidelity 2X Master Mix (New England Biolabs) and forward primer 5’-cgggatcccaaatgacgcaactcactga-3’ and reverse primer 5’-cagaattcctaagctcgaagcaatgcaa-3’ (N-terminal V5-tag) or forward primer 5’-tcggatccaccatgtctcaaatgacgca-3’ and reverse primer 5’- tagaattcagctcgaagcaatgcaagttcaat-3’ (C-terminal V5-tag). PCR products were inserted between the BamHI and EcoRI restriction sites of the V5-pCDNA3.1(+) or pCDNA3.1(+)-V5 plasmids.

The V5-M_N3Q_-∆20_ct_ mutant, lacking the last 20 amino acids of the M protein, was generated by PCR using forward primer 5ʹ-cgggatcccaaatgacgcaactcactga-3ʹ and reverse primer 5ʹ-tagaattcttacttatatctatggtaaatgg-3ʹ. V5-M_N3Q_-_211_DxE_213_A and V5-M_N3Q_-_199_KxGxYR_204_A mutants were generated by fusion PCR. The first PCR was performed with forward primer 5ʹ-cgggatcccaaatgacgcaactcactga-3ʹ and reverse primer 5ʹ-caagtgcaatagccgccgtaataggcggactcc-3ʹ for _211_DxE_213_A or reverse primer 5ʹ-cggactagcagcattagctgccgcatatctatggtaaatggca-3ʹ for _199_KxGxYR_204_A. The second PCR was performed with forward primer 5ʹ-acggcggctattgcacttgcattgcttcgagct-3ʹ for _211_DxE_213_A or forward primer 5ʹ-agatatgcggcagctaatgctgctagtccgcctattacggcgg-3ʹ for _199_KxGxYR_204_A and reverse primer 5ʹ-cctactcagacaatgcgatg-3ʹ. Fusion PCRs were performed with forward primer 5ʹ-cgggatcccaaatgacgcaactcactga-3ʹ and reverse primer 5ʹ-cagaattcctaagctcgaagcaatgcaa-3ʹ for both constructs. All PCR products were inserted between the BamHI and EcoRI restriction sites of the V5-pCDNA3.1(+) vector. Plasmid sequences were verified by Sanger sequencing.

### Plasmid construction for MERS-CoV E and N protein expression

The coding sequences of the MERS-CoV E and N proteins, obtained from an infected patient hospitalized in Lille, France, were cloned between the BamHI and EcoRI restriction sites of a pCDNA3.1(+) vector, with a C-terminal HSV-tag coding sequence. As previously described [27], cDNA obtained after reverse transcription of RNA extracted from a blood sample of an infected patient was used for amplification of the protein-coding sequences. First, amplification was performed using forward primer 5ʹ-atgttaccctttgtccaaga-3ʹ and reverse primer 5ʹ-ttaaacccactcgtcaggtg-3ʹ for E and with forward primer 5ʹ-atggcatcccctgctgcacc-3ʹ and reverse primer 5ʹ-atcttgttactttgagtgac -3ʹ for N. To insert the sequence between the BamHI and EcoRI restriction sites in the pCDNA3.1(+) expression vectors, amplification was performed with forward primer 5ʹ-tcggatccaccatgttaccctttgtccaagaacgaa-3ʹ and with the reverse primer 5ʹ-ccgaattcaacccactcgtcaggtggtagagg-3ʹ for E and with forward primer 5ʹ- tcggatccaccatggcatcccctgc -3ʹ and with the reverse primer 5ʹ- tggaattcatcagtgttaacatcaatcattgg -3ʹ for N.

### Plasmid construction for MERS-S protein

The pCAGGS-MERS-S, containing a codon-optimized sequence of MERS-CoV S, was kindly provided by Gary Whittaker. This sequence was subsequently inserted between the BamHI and EcoRI restriction sites in a pCDNA3.1(+) expression vector.

### Cells

Huh-7 cells were used for all experiments, with the exception of Huh-7-DPP4-knockout (KO) cells when also the incorporation of the S protein was assessed in the VLPs. Huh-7 and Huh-7-DPP4-KO cells were maintained in DMEM supplemented with 10% fetal calf serum (FCS) and 1% Glutamax.

### CRISPR-Cas9 knockout of DPP4 in Huh-7 cells

The Huh-7-DPP4-KO cells were generated by CRISPR-Cas9 knockout of DPP4 in Huh-7 cells. To generate the sgRNA expression vector, the oligos 5ʹ-caccgaagagaataaactgccatc-3ʹ and 5ʹ-aaacgatgggcagtttattctcttc-3ʹ were annealed and cloned into the pSpCas9 vector after BbsI restriction. Huh-7 cells seeded into six-well plates were co-transfected using TransIT®-LT1 Transfection Reagent (Mirus Bio) with 1 µg of sgRNA expression vector and 50 ng of pPURO to confer resistance to puromycin. Cells were selected with 5 µg/ml of puromycin for 5 days. Then clones were isolated from the DPP4-KO population and one clone was selected for the VLP assay.

### VLP assay

Huh-7 or Huh-7-DPP4-KO cells were transferred to 100 mm dishes at a concentration of 2 × 10^6^ cells per dish 16 h before transfection. To test the minimal requirements for VLP formation, Huh-7 or Huh-7-DPP4-KO cells were transfected with 2 µg of M-V5- or V5-M-encoding plasmids, 1 µg of E-HSV-encoding plasmid, and 3 µg of S-encoding plasmid, either alone or in combination with each other, using TransIT®-LT1 Transfection Reagent (Mirus Bio). Empty pcDNA3.1(+) vector was used to complete the total amount of transfected DNA to 6 µg if necessary. To test the effect of mutant M proteins on the basic VLP formation ability, Huh-7 cells were co-transfected with 2 µg of plasmid encoding for V5-M_N3Q_ (wild-type) or mutant M protein (V5-M_N3Q_-∆20_ct,_ V5-M_N3Q_-_211_DxE_213_A and V5-M_N3Q_-_199_KxGxYR_204_A or the double mutant V5-M_N3Q_-_199_KxGxYR_204_A-_211_DxE_213_A) and 2 µg of E-HSV-encoding plasmid. To test the secretion of wild-type or mutant M proteins upon single expression, cells were co-transfected with 2 µg of the plasmids encoding wild-type or mutant M proteins and 2 µg of the empty pCDNA3.1(+) vector. When the N incorporation had to be assessed, lower E concentrations were used, leading to a combination of 2 µg V5-M_N3Q_-encoding plasmid, 0.25 µg E-HSV-encoding plasmid, and 5 µg of N-HSV encoding plasmid. S incorporation was assessed using 3 µg of S-encoding plasmid.

Forty-eight hours post-transfection, the supernatant (10 ml) was collected, centrifuged (150 g, A-4–62 rotor, Eppendorf, 5 min, 4 °C) and filtered through a 0.45 µm filter. Cells were rinsed twice with cold phosphate buffered saline (PBS) and lysed with RIPA buffer (50 mM Tris–HCl pH 7.5, 150 mM NaCl, 1% NP-40, 0.5% DOC, 0.1% SDS) containing cOmplete™ protease inhibitor cocktail (Roche) for 1 h at 4 °C. Supernatant samples were loaded on a 20% sucrose cushion (2 ml) and centrifuged at 4 °C for 3 h at 154,000 g (SW41Ti rotor, Beckman). Cell lysates were centrifuged at 18,000 g (10 min, 4 °C, FA-45-30-11 rotor, Eppendorf) and lysates were stored at − 20 °C until western blot analysis. After ultracentrifugation of the supernatant, pellets were resuspended in 200 µl TN buffer (20 mM Tris–HCl pH 7.4, 100 mM NaCl) and stored at − 80 °C until western blot analysis.

### Western blot analysis

VLP pellets in TN buffer and cell lysates were resuspended in Laemmli loading buffer and separated on a 12% (M, N and E) or 8% (S) polyacrylamide gel by SDS-PAGE. Next, proteins were transferred to a nitrocellulose membrane (Amersham), and the membranes were subsequently blocked for 1 h at RT in 5% (w/v) non-fat dry milk in PBS with 0.1% (v/v) Tween-20. For the detection of the V5-tagged M protein, membranes were incubated overnight at 4 °C with monoclonal anti-V5 antibodies (ThermoFisher Scientific) in 5% (w/v) non-fat dry milk in PBS with 0.1% (v/v) Tween-20. For the detection of untagged M, polyclonal rabbit anti-MERS-CoV-M antibodies were used (Proteogenix). For the detection of E-HSV, overnight incubation of the membranes occurred with polyclonal goat anti-HSV antibodies (Abcam), for N-HSV with polyclonal goat anti-HSV antibodies (Abcam) or polyclonal rabbit anti-N antibodies (Invitrogen), whereas membranes were incubated overnight with polyclonal rabbit anti-spike antibodies (Sino Biological) for the detection of the S protein. After being washed three times with PBS with 0.1% (v/v) Tween-20, membranes were incubated for 1 h at RT with HRP-labeled goat-anti mouse IgG antibodies (V5-tagged M protein), donkey anti-sheep IgG antibodies (E or N protein) or goat anti-rabbit IgG antibodies (S, N or untagged M proteins) (Jackson ImmunoResearch), after which membranes were washed three times. Proteins were visualized by enhanced chemiluminescence (Pierce™ ECL, ThermoFisher Scientific).

### Electron microscopy

Huh-7-DPP4-KO cells were transferred to 100 mm dishes at a concentration of 2 × 10^6^ cells per dish 16 h before transfection. Cell medium was replaced by DMEM with 2% FCS. For the detection of the M + E_high_ + S VLPs, cells were co-transfected with 2 µg of plasmid encoding for V5-M_N3Q_, 1 µg of E-HSV-encoding plasmid, and 3 µg of S-encoding plasmid using the TransIT®-LT1 Transfection Reagent. For the detection of the M + E_low_ + N + S VLPs, cells were co-transfected with 2 µg of plasmid encoding for V5-M_N3Q_, 0.25 µg of E-HSV-encoding plasmid, 5 µg of N-HSV-encoding plasmid, and 3 µg of S-encoding plasmid using the TransIT®-LT1 Transfection Reagent. Forty-eight hours post-transfection, the supernatant was collected for VLP precipitation as described above. After ultracentrifugation of the supernatant (141,000 g, SW28Ti rotor, Beckman), the pellet was resuspended in 50 µl PBS and fixed by addition of 50 µl 8% PFA. Formvar/carbon-coated nickel grids were deposited on a drop of samples during 5 min and rinsed two times on a drop of water. The negative staining was then performed with three consecutive contrasting steps using 2% uranyl acetate (Agar Scientific, Stansted, UK), before analysis under the transmission electron microscope (JEOL 1011, Tokyo, Japan).

### Reverse genetics

The bacterial artificial chromosome (BAC) containing a full-length infectious MERS-CoV cDNA, referred to as wild-type BAC in the present paper, was kindly provided by Dr. F. Almazan and Dr. L. Enjuanes [44]. Construction of BAC M mutants was done using an intermediate pCDNA3.1(+) plasmid, containing nucleotides 35,422 to 261 of the circular wild-type BAC (and hence containing the E–M–N structural proteins), between a SanDI (KflI) and an SfiI restriction site, naturally present in the wild-type BAC. The _199_KxGxYR_204_A mutant was generated by fusion PCR using the intermediate pCDNA3.1(+)-E-M–N plasmid as a template. The first PCR was performed with forward primer 5ʹ- aagggtcccgtgtagaggctaatccatt-3ʹ and reverse primer 5ʹ- cggactagcagcattagctgccgcatatctatggtaaatggca-3ʹ. The second PCR was performed with forward primer 5ʹ-agatatgcggcagctaatgctgctagtccgcctattacggcgg-3ʹ and reverse primer 5ʹ- gtggcccgggcggccgcaaggggttcgc-3ʹ. Fusion PCR was performed with the forward primer of the first PCR reaction and the reverse primer of the second PCR reaction. The fusion PCR product was inserted between the SanDI and SfiI restriction sites of the pCDNA3.1(+) vector and the sequence of the full fragment was verified by Sanger sequencing. Next, this fragment was brought into the wild-type BAC by means of restriction digest and ligation. A similar approach was used for the _211_DxE_213_A construct. To construct the intermediate pCDNA3.1(+)-E-M_DxE_-N vector, the first PCR was performed with forward primer 5ʹ-aagggtcccgtgtagaggctaatccatt -3ʹ and reverse primer 5ʹ-caagtgcaatagccgccgtaataggcggactcc-3ʹ, the second PCR with forward primer 5ʹ-ttacggcggctattgcacttgcattgcttcgagctta-3ʹ and reverse primer 5ʹ-gtggcccgggcggccgcaaggggttcgc-3ʹ, and fusion PCR was performed with forward primer 5ʹ-aagggtcccgtgtagaggctaatccatt -3ʹ and reverse primer 5ʹ-gtggcccgggcggccgcaaggggttcgc-3ʹ. The fusion PCR product was inserted between the SanDI and SfiI restriction sites of the pCDNA3.1(+) vector and the sequence of the full fragment was verified by Sanger sequencing.

### Co-immunoprecipitation

Huh-7 cells were seeded in six-well plates at a concentration of 0.4 × 10^6^ cells per well 16 h before transfection. Cells were co-transfected with 0.5 µg of plasmid encoding for V5-M_N3Q_ (wild-type) or mutant M protein (V5-M_N3Q_-∆20_ct,_ V5-M_N3Q_-_211_DxE_213_A and V5-M_N3Q_-_199_KxGxYR_204_A or the double mutant V5-M_N3Q_-_199_KxGxYR_204_A-_211_DxE_213_A) and 0.5 µg of E-HSV-encoding plasmid (co-IP M–E) or 0.5 µg of M_N3Q_-HSV (co-IP M–M) using the TransIT®-LT1 Transfection Reagent. Empty vectors were used for the control conditions or to complete the total amount to 1 µg if necessary. For M–E co-IP, cells were washed with PBS and proteins were cross-linked by formaldehyde treatment (0.8%, 10 min at RT). Afterward, cells were washed twice with 500 mM Tris–HCl pH 7.4 in PBS and once with cold PBS before lysis. For M–M co-IP, no cross-linking was performed and cells were washed twice with cold PBS before lysis. Lysis was performed in RIPA buffer (50 mM Tris–HCl pH 7.5, 150 mM NaCl, 1% NP-40, 0.5% DOC, 0.1% SDS) for both M–E and M–M co-IP experiments for 1 h at 4 °C. After centrifugation (18,000 g, 10 min, 4 °C, FA-45–30-11 rotor, Eppendorf), the lysates were precleared by incubation with protein G sepharose beads for 45 min at 4 °C. The precleared lysates were subsequently divided into two fractions, one for incubation with goat polyclonal anti-V5 antibodies (Abcam), the other for incubation with rabbit anti-HSV antibodies (Novus), this for 3 h at 4 °C. Meanwhile, protein G sepharose beads were blocked in 1% BSA in PBS, after which they were washed two times with RIPA buffer before addition of the lysate–antibody suspensions. After 1 h incubation at 4 °C, beads were washed five times with RIPA buffer containing cOmplete™ protease inhibitor cocktail, after which the proteins were eluted by addition of Laemmli buffer containing 2-mercaptoethanol and heating for 10 min at 95 °C. Eluates were analyzed by western blot analysis using the same antibodies as described above for the VLP analysis.

### Image J analysis

Quantification of western blots band was performed by Image J and its band quantification function. To compensate for differences in expression levels, the ratio of VLP secretion was calculated (= M_medium_/M_lysate + medium_) and plotted relative to wild-type M for all mutants. For Co-IP experiments, the co-IP and IP signals were calculated relative to the wild-type M and the average co-IP signal (= (anti-V5 signal + anti-HSV signal)/2) was normalized for the average IP signal (= (anti-HSV signal + anti-V5 signal)/2) to take into consideration the dependency of the co-IP signal on both the efficiency of the immunoprecipitation and the total precipitable amount of proteins. As the E-HSV signal in the co-precipitated samples was too weak, only the co-precipitated V5-M signal was calculated and plotted relative to the average IP signal for the M–E co-IP experiments.

### Immunofluorescence and confocal microscopy

Huh-7 cells were seeded on coverslips in 24-well plates at a concentration of 0.08 × 10^6^ cells per well 16 h before transfection. Cells were transfected with a total of 250 ng plasmid, encoding for V5-M_N3Q_ or mutant M protein all or not in combination with E-HSV-encoding plasmid (co-localization M–E) or M_N3Q_-HSV-encoding plasmid (co-localization M–M) using the TransIT®-LT1 Transfection Reagent. Sixteen hours post-transfection, cells were fixed with 3% formaldehyde in PBS for 20 min at RT. After permeabilization with 0.1% Triton-X100 in PBS for 3 min at RT, cells were incubated with 10% normal horse serum for 15 min at RT. V5-tagged M proteins were visualized by incubation with monoclonal anti-V5 antibodies in 10% normal horse serum (ThermoFisher Scientific), followed by incubation with cyanine-3-conjugated donkey anti-mouse IgG secondary antibodies. HSV-tagged proteins were labeled with polyclonal rabbit anti-HSV antibodies (Abcam) in 10% normal horse serum, followed by Alexa Fluor® 488-conjugated donkey anti-rabbit IgG antibodies. The trans-Golgi network was visualized by incubation with polyclonal sheep anti-human TGN antibodies (BioRad), followed by incubation with Alexa Fluor® 647-conjugated donkey anti-sheep IgG antibodies. Nuclei were visualized with 1 µg/ml of 4ʹ,6-diamidino-2-phenylindole (DAPI), and coverslips were mounted in Mowiol® mounting medium. Images were acquired using a laser scanning confocal microscope LSM 880 (Zeiss) using a 63 × oil immersion objective. Pearson’s correlations coefficients were calculated using the JACoP plugin of ImageJ.

### HiBiT-based assay for the quantification of plasma membrane expression levels

Huh-7 cells were seeded at a density of 1.3 × 10^4^ in white 96-well microplates (Thermo Fisher Scientific). The next day, cells were transfected with plasmids encoding for HiBiT-M_N3Q_, HiBiT-M_N3Q_-∆20_ct,_ HiBiT-M_N3Q_-_211_DxE_213_A and HiBiT-M_N3Q_-_199_KxGxYR_204_A at a concentration of 100 ng/well using the TransIT®-LT1 Transfection Reagent. Sixteen hours post-transfection, the medium was removed and 50 µl DMEM without FCS was added to each well. To assess the plasma membrane expression, a mix containing 50 µl extracellular buffer, 0.5 µl LgBiT, and 1 µl extracellular substrate was added per well (Nano-Glo® HiBiT Extracellular Detection System-Promega). The total protein expression levels were assessed by adding a mix containing 50 µl lytic buffer, 0.5 µl LgBiT, and 1 µl lytic substrate (Nano-Glo® HiBiT Lytic Detection System-Promega). Luciferase activity was measured by the use of a Tristar LB941 luminometer (Berthold Technologies). For each construct, the ratio plasma membrane signal/total protein signal was calculated and plotted relative to the wild-type M. For each condition, duplicate wells were taken and experiments were repeated at least three times.

### BAC transfection

Huh-7 cells were transferred to 24-wells 16 h before transfection with 1.2 µg of the wild-type or mutated BAC constructs using TransIT®-LT1 Transfection Reagent (Mirus Bio). Twenty-four hours post-transfection, duplicate wells of cells were collected for each construct and RNA was extracted using the NucleoSpin® RNA Plus kit (Macherey–Nagel®), according to the manufacturer’s instructions. For each construct, also cells lysates were collected at 48 h post-transfection in Laemmli buffer and heated at 95 °C for 30 min to visualize the M, E, N, and S structural proteins by western blotting. The antibodies used for immunoblotting have been described above, except for the anti-E protein antibodies for which polyclonal rabbit MERS-CoV-E antibodies were used (GeneTex). For the remaining wells, medium was changed 24 h post-transfection, and 4 days post-transfection, supernatants were collected for infectivity titrations and cells were fixed for immunofluorescence staining, using primary polyclonal rabbit anti-MERS-CoV-M antibodies (Proteogenix) to visualize the M localization for all constructs.

### qPCR

MERS-CoV genomes were quantified by real-time quantitative RT-PCR. Briefly, cDNA was obtained by reverse transcription of RNA using the high-capacity cDNA reverse transcription kit (Life Technologies). MERS-CoV genomes were measured by quantitative RT-PCR using a Quant-studio3. Amplification was performed using a pair of specific primers (5ʹ- caaaaccttccctaagaaggaaaag-3ʹ and 5ʹ-gctcctttggaggttcagacat-3ʹ) as well as a probe (5ʹ-FAM-accaaaaggcaccaaaagaagaatcaacagacc-3ʹ) specific for MERS-CoV-N protein sequence. A standard curve was prepared with RNA generated by transcription of a pCDNA3.1 plasmid containing the N protein-coding sequence using the kit. Serial dilutions of these RNAs were used in parallel during the reverse transcription of the cellular RNA.

### Infectivity titration

Four days after BAC transfection, cell supernatants were collected and the amount of infectious virus was determined by infectivity titration. Therefore, Huh-7 cells, seeded in 96-well plates, were inoculated with 100 µl of 1/10 serially diluted supernatants (ranging from 10^–1^ to 10^–8^). Cells were incubated with the virus dilutions for 5 days at 37 °C and 5% CO_2_. Then the 50% tissue culture infectious dose (TCID_50_) was determined by assessing the CPE in each well by light microscopy and the 50% end point was calculated according to the method of Reed and Muench.

## Results

### MERS-CoV VLP formation minimally requires both M and E proteins and does not need the N-glycans on the M protein

MERS-CoV VLP formation has been described upon co-transfection experiments in HEK293T cells [34, 37]. Therefore, in a first attempt to produce VLPs, HEK293T cells were co-transfected with plasmids encoding the different structural proteins, either alone or in combination. However, in our experimental conditions, we did not manage to reliably assess the VLP formation capacity in these cells, notably because the intracellular expression levels of the proteins varied greatly in the different conditions (single vs multi-transfection) (Fig. S1 and supplementary information). In Huh-7 cells on the contrary, a stable and uniform expression level was noticed for all proteins in all conditions over time in our hands, and hence these cells were used to further assess the MERS-CoV VLP formation.

**Fig. 1:**
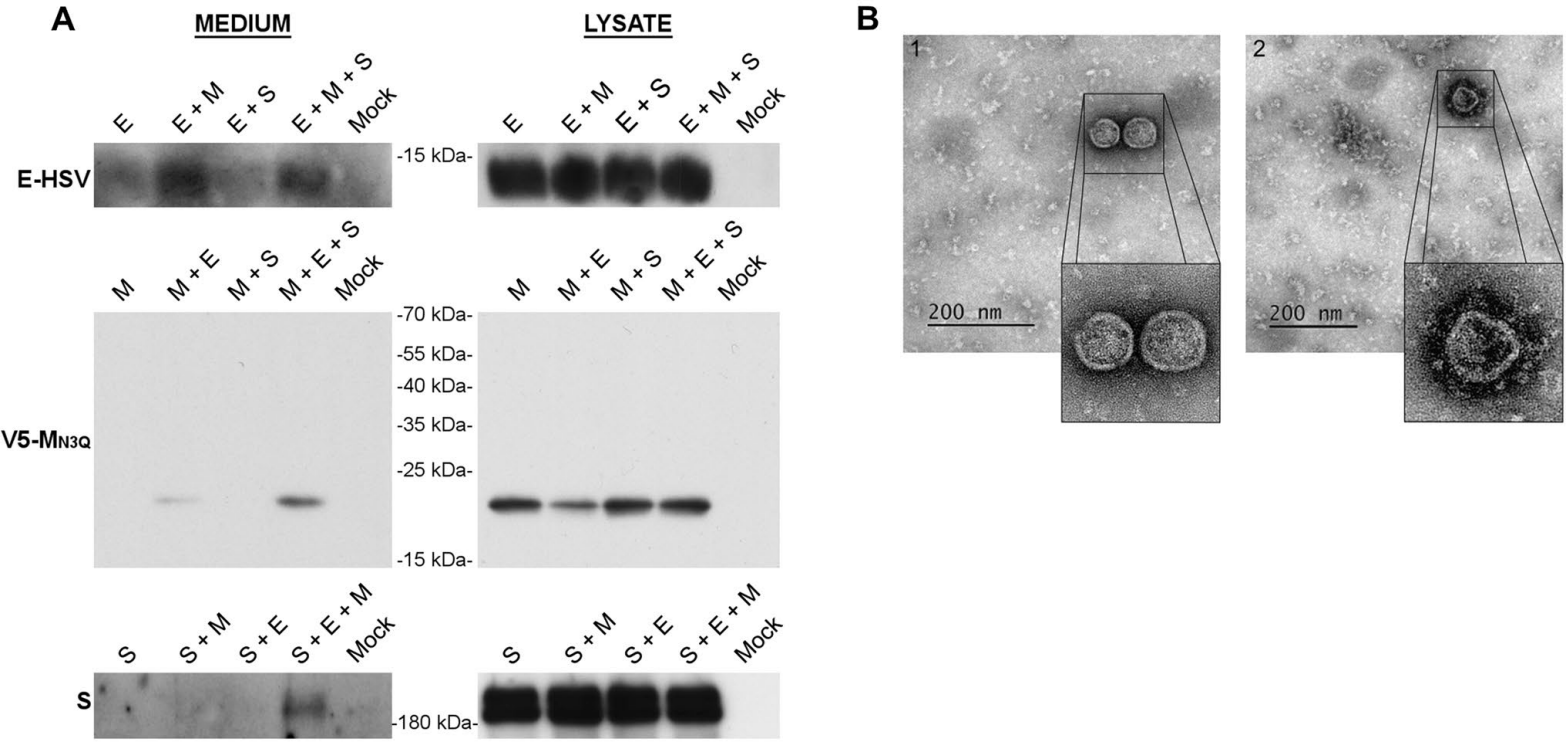
MERS-CoV VLP formation in Huh-7-DPP4-KO cells. **A** Representative immunoblot images showing the MERS-CoV E, M, and S proteins in the pelleted supernatant (= medium) and the expression levels of the proteins in the cells (= lysate) 48 h after single or combined transfection of 1 µg of E-HSV-, 2 µg of unglycosylated V5-M_N3Q_-, and 3 µg of S-encoding plasmids in 2 × 10^6^ Huh-7-DPP4-KO cells. **B** EM images of the pelleted supernatant to visualize the formation of VLPs 48 h after co-transfection of the abovementioned concentrations of E-HSV-, V5-M_N3Q_-, and S-encoding plasmids in Huh-7-DPP4-KO cells

**Fig. 2:**
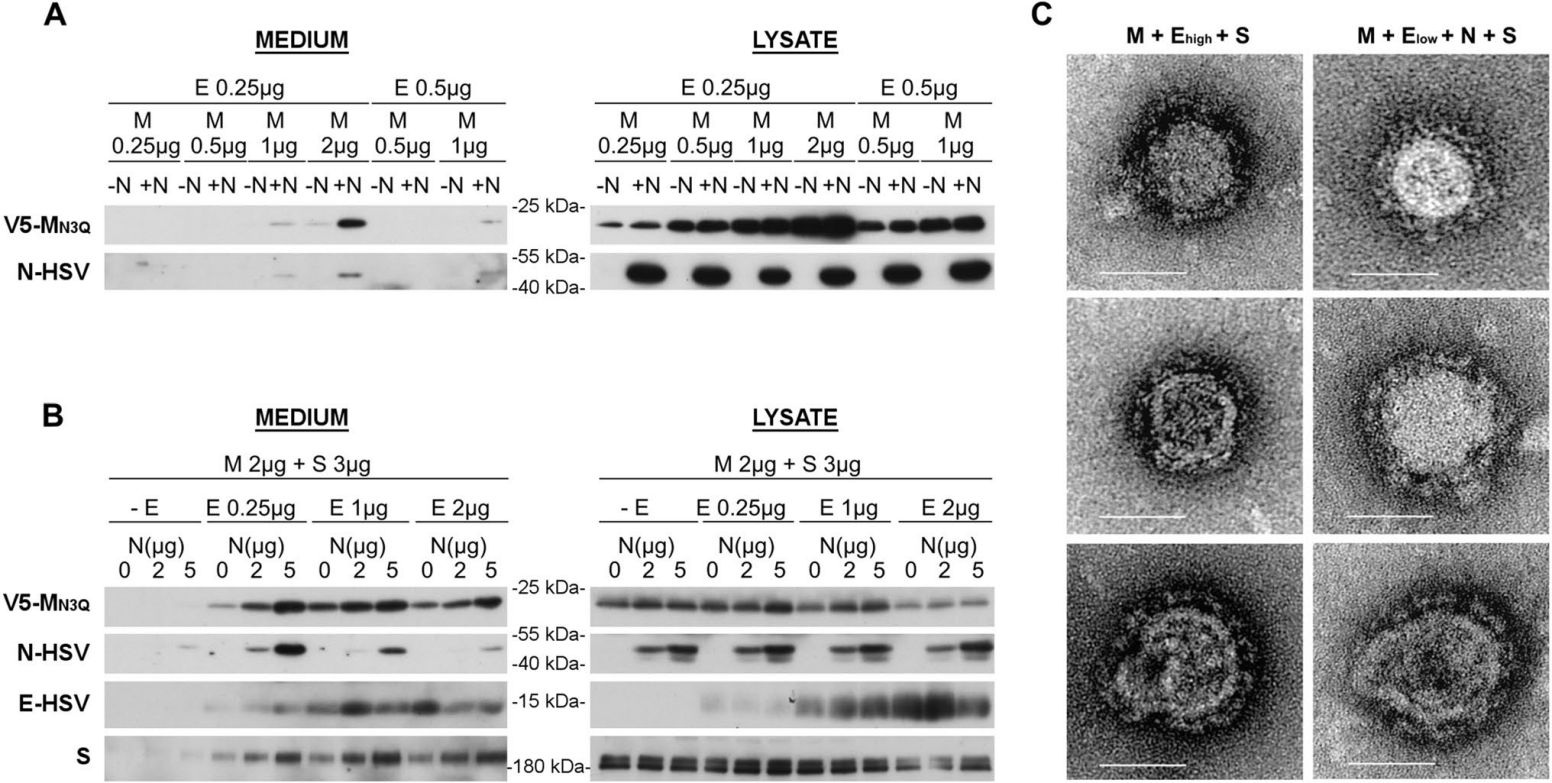
Effect of N co-expression on MERS-CoV VLP formation. **A** Extracellular release and expression levels of MERS-CoV M and N proteins upon co-transfection of 2 µg N-HSV-encoding plasmid with various, suboptimal concentrations of V5-M_N3Q_- and E-HSV-encoding plasmids. **B** Representative immunoblot images showing the extracellular release (= medium) and expression levels (= lysate) of MERS-CoV M, N, E, and S proteins upon co-transfection of fixed amounts of V5-M_N3Q_ and S-encoding plasmids (2 and 3 µg, respectively) with different concentrations of E-HSV (0, 0.25, 1, or 2 µg) and N-HSV-encoding plasmids (0, 2 or 5 µg) in 2 × 10^6^ Huh-7-DPP4-KO cells. **C** EM images of the pelleted supernatant to visualize the M + E_high_ + S VLPs (left panel) and the M + E_low_ + N + S VLPs (right panel). Scale bar represents 50 nm

After optimization of the VLP production assay in Huh-7 cells (described in more detail in supplementary information), we found that co-expression of E and M proteins was minimally required for VLP formation and that N-glycosylation of M was not required (Fig. 1A, Fig. S2A and B). Moreover, it was noticed that addition of a C-terminal V5-tag artificially increased the release of the single-expressed M protein (Figure S2B and C). Consequently, an N-terminally V5-tagged M protein was used for all the subsequent experiments, mostly in combination with the N3Q mutation. The latter allows a better visualization and more reliable quantification of the M protein on western blot (only 1 band instead of 3) as described before [27]. As expected, expression of S did not enhance the formation of VLPs but the protein was incorporated into VLPs when co-expressed with E and M (Fig. 1A). However, to detect the release of S-decorated VLPs, it was necessary to prevent their binding to the cell surface using a DPP4 deficient cell line (Fig. S2A and Fig. 1A). Therefore, a DPP4-KO-Huh-7 cell line was generated for this purpose by CRISPR-Cas9-mediated gene inactivation. Secretion of the E protein was visible in all conditions, but this secretion was enhanced in the conditions where M was present. The E signal was generally very weak on blot and required longer exposure time than M proteins, suggesting that only very low amounts of E proteins were present in the VLPs. To assure that western blot detection of the proteins resulted from VLP formation, and not from the increased non-specific secretion of these proteins, electron microscopy (EM) imaging was performed on the pelleted supernatant, revealing the presence of assembled virus-like structures either without (Fig. 1B, 1) or with (Fig. 1B, 2) S incorporation. The size of S-bearing VLPs was very heterogenous ranging from 72 to 90 nm.

CoVs typically generate a nested set of subgenomic mRNAs for the translation of their structural and accessory proteins. This implicates that not all structural proteins are present in equal amounts during CoV infection. Therefore, we assessed if changes in M and E concentrations would impact M + E VLP formation. It was found that VLPs were only detectable at higher M concentrations (2 µg/2 × 10^6^ cells), whereas lowering the E concentration down to 0.5 µg was sufficient to clearly see the VLP-associated M secretion (Fig. S3). Higher E concentrations had no impact on VLP formation. Taken together, these data show that M and E are the minimal requirements for MERS-CoV VLP formation, which can be detected by western blot analysis on the pelleted supernatant of Huh-7 cells that co-express the MERS-CoV V5-M_N3Q_ and E proteins.

### High E concentrations generate nucleocapsid-empty VLPs, whereas low E concentrations generate VLPs that contain all four structural proteins

For other CoVs [29, 31, 39], N protein co-expression has been required to assure efficient VLP formation, and it has been hypothesized that N co-expression might be especially important to drive VLP formation in less abundant expression conditions [31]. Therefore, the effect of MERS-CoV N co-expression (2 µg/2 × 10^6^ cells) was first tested in the Huh-7-based VLP assay using suboptimal M + E conditions, by lowering the E and/or M concentration (Fig. 2A). N co-expression clearly enhanced the VLP formation at lower E concentration (0.25 µg), but not at lower M concentrations, for which at least 2 µg was still required for VLP formation.

To have a broader view on the effect of N co-expression on VLP formation, fixed M and S concentrations (2 and 3 µg/2 × 10^6^ cells, respectively) were combined with varying E and N concentrations and VLP formation was assessed (Fig. 2B). These results showed that also in higher E concentration conditions, N co-expression dose dependently improved the VLP signal, at least for the M and S proteins, as the effect on the E protein was less clear. The N incorporation was dose dependent as well (the more N the better the N incorporation). Interestingly, N incorporation into VLPs decreased when E concentration increased, indicating that raising the E concentration increased the odds for nucleocapsid-empty particles to be formed.

Taken together, two different kinds of VLPs seemed to arise by changing the E concentration—first, basic M + E VLPs that were easily detectable in the pelleted medium when using higher E concentrations, but which did not very well incorporate the N protein (called M + E_high_ VLPs in the rest of the manuscript); second, VLPs that arose in lower E concentration conditions and showed a good N incorporation (called M + E_low_ + N VLPs). For both VLPs, S had no effect on the VLP formation and was incorporated when co-expressed. Comparison of the M + E_low_ + N + S VLPs with the M + E_high_ + S VLPs by EM imaging did not show obvious morphological differences between both VLPs (Fig. 2C).

### Deletion of the C-terminal 20 amino acids and mutation of the _199_KxGxYR_204_ motif severely impair the M + E_high_ VLP formation

**Fig. 3:**
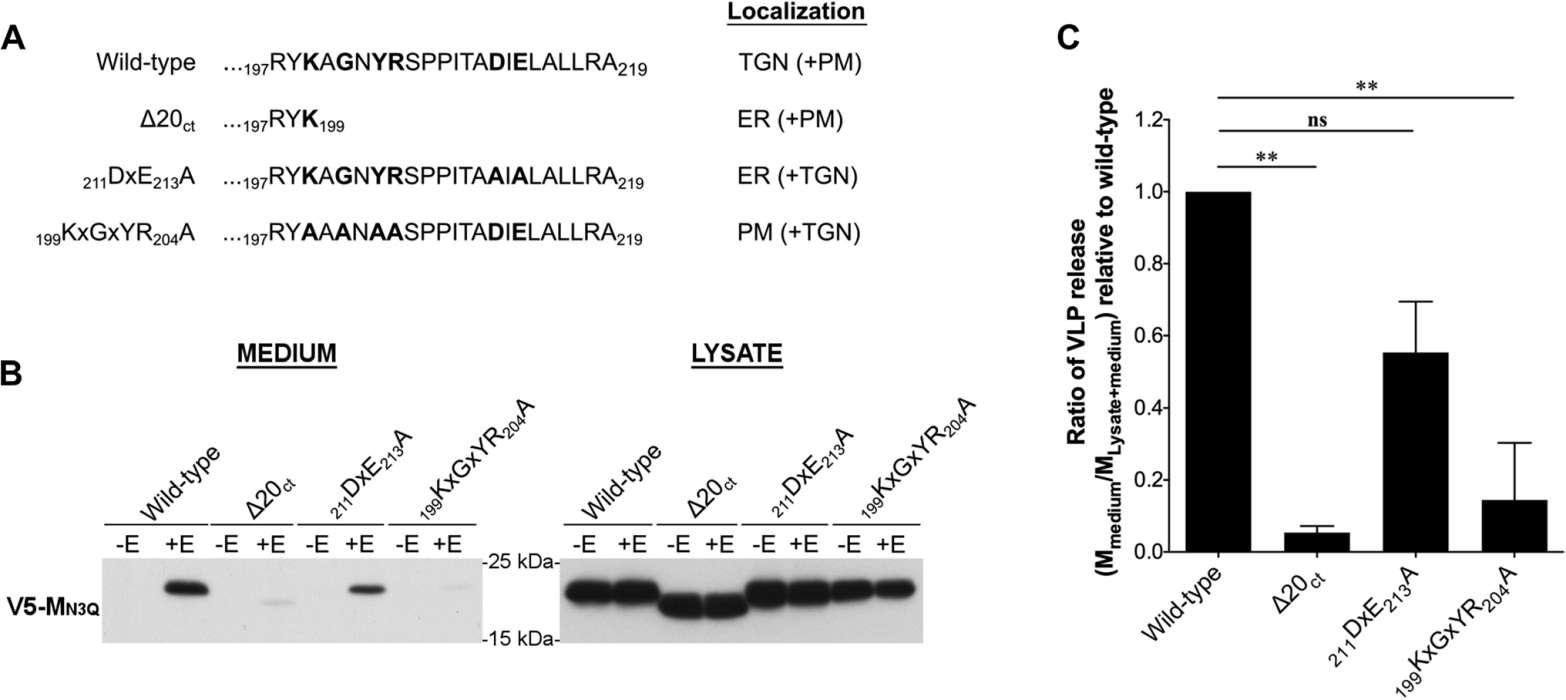
Effect of ∆20_ct_, _211_DxE_213_A, and _199_KxGxYR_204_A mutations on M + E_high_ VLP formation. **A** Schematic representation of the M C-tail amino acid composition of the described mutants and their main and minor (brackets) subcellular localization sites. **B** Representative immunoblot images showing the wild-type and mutant M proteins in the pelleted supernatant (= medium) and the expression levels of the proteins in the cells (= lysate) 48 h after transfection of 2 × 10^6^ Huh-7 cells with V5-tagged wild-type MERS-CoV M, ∆20_ct_, _211_DxE_213_A, or _199_KxGxYR_204_A-encoding plasmids (2 µg), alone or in combination with E-HSV-encoding vector (2 µg). **C** The VLP secretion ratio (= M_medium_/M_lysate + medium_) of all mutants was calculated and expressed relative to wild-type M. Data represent the mean + standard deviation from at least four independent experiments. Significant differences (*P ≤ 0.05) were assessed by the Kruskal––Wallis test with Dunn’s correction for multiple comparisons

To assess if the subcellular localization of the M protein is important for virus assembly, three MERS-CoV M mutants that show mislocalization were used, including ∆20_ct_, _211_DxE_213_A, and _199_KxGxYR_204_A mutants. The main subcellular localization of these three mutants was documented before [27]. Briefly, at steady state, the wild-type protein is mainly detected in the TGN, whereas the ∆20_ct_ and _211_DxE_213_A mutants are found in the ER. The _199_KxGxYR_204_A mutant is strongly detected at the cell surface. Figure 3A summarizes these steady-state localization sites. Since M proteins are not confined to only their main localization site, minor localization sites are specified between brackets and also indicated in Figure S4.

For all MERS-CoV M mutants, it was decided to initially study their effect on assembly using the least complex, basic M + E_high_ VLP assay. Moreover, it was decided to keep equimolar concentrations of M and E protein-coding plasmids in all experiments to ensure visibility of the E protein, if necessary. More precisely, for each vector, 125 ng was added per 24-well (0.08 × 10^6^ cells) for immunofluorescence staining, 500 ng was added per 6-well (0.4 × 10^6^ cells) for immunoprecipitation experiments, and 2 µg was added per 100 mm dish (2 × 10^6^ cells) for M + E_high_ VLP studies.

To verify that none of the mutants affected the non-specific secretion of the single-expressed M protein, transfection was performed either without or with MERS-CoV E for all mutants and the wild-type M protein. None of the mutants induced non-specific M secretion. The M + E_high_ VLP production was greatly affected for the ∆20_ct_ and _199_KxGxYR_204_A mutants, whereas _211_DxE_213_A-VLPs were still formed (Fig. 3B and C).

### ∆20_ct_ and _199_KxGxYR_204_A mutants show a reduced M–E interaction, whereas there is a suboptimal M–M interaction with the _211_DxE_213_A mutant

**Fig. 4:**
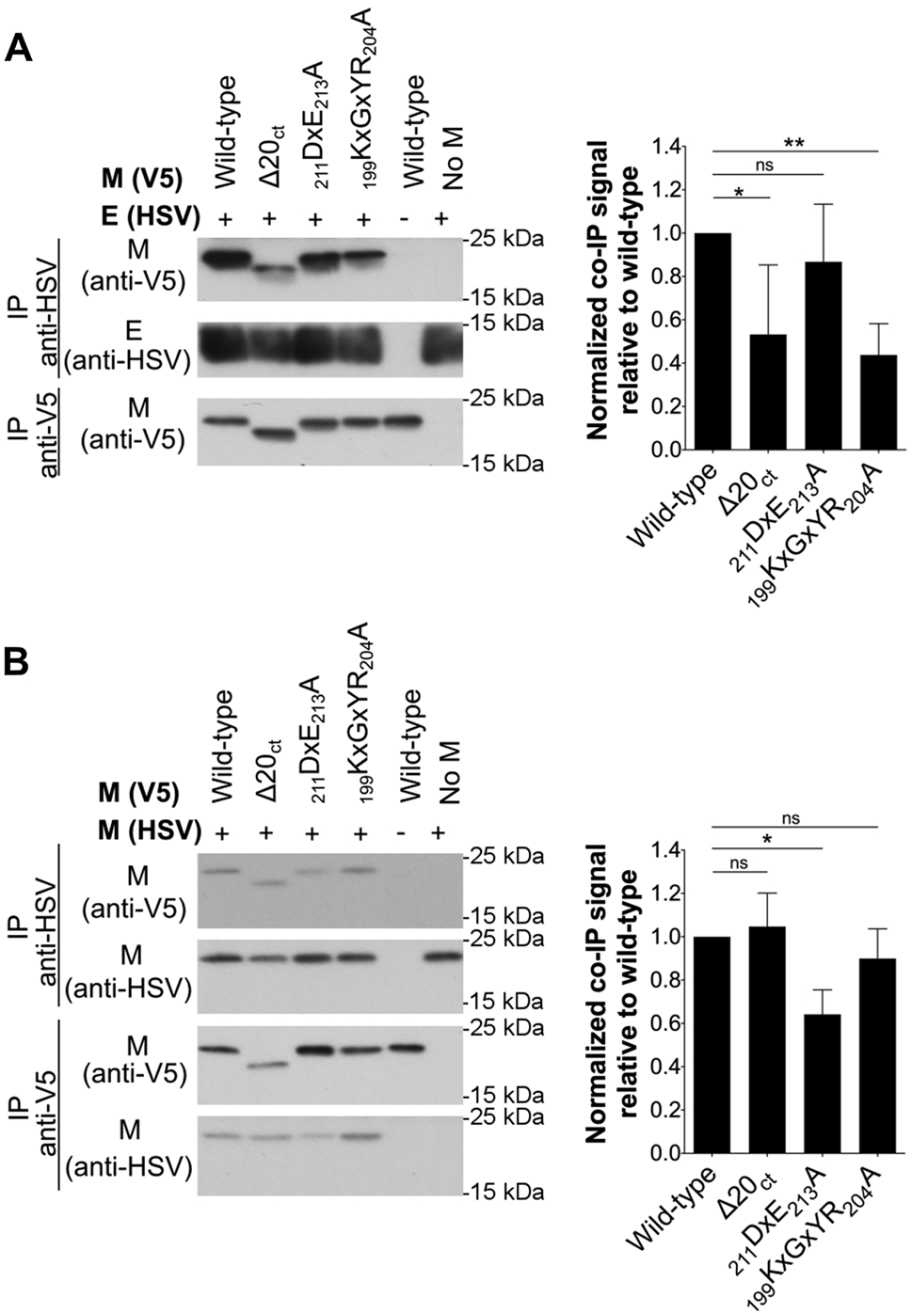
Effect of ∆20_ct_, _211_DxE_213_A, and _199_KxGxYR_204_A mutations on M–E and M–M interactions. **A** V5-tagged wild-type and ∆20_ct_, _211_DxE_213_A, or _199_KxGxYR_204_A mutant M-encoding plasmids were co-expressed with equimolar concentrations of E-HSV (0.5 µg of each/0.4 × 10^6^ Huh-7 cells) and an immunoprecipitation was performed with both anti-V5 and anti-HSV antibodies 24 h post-transfection after cross-linking with 0.8% formaldehyde. Left: representative immunoblot image of the M–E co-IP experiment. Co-precipitated E-HSV proteins (IP anti-V5) were undetectable and hence not shown. Right: the normalized co-IP signal was plotted relative to the wild-type M protein for all mutants. Data result from four (∆20_ct_) or six (_211_DxE_213_A and _199_KxGxYR_204_A mutant) independent experiments and significant differences, as assessed by the Kruskal–Wallis test with Dunn’s correction for multiple comparisons, are indicated with an asterisk (*P ≤ *0.05). **B** V5-tagged wild-type and ∆20_ct_, _211_DxE_213_A, and _199_KxGxYR_204_A mutant M-encoding plasmids were co-expressed with equimolar concentrations of M_N3Q_-HSV (0.5 µg of each/0.4 × 10^6^ Huh-7 cells) and an immunoprecipitation was performed with anti-V5 and anti-HSV antibodies 24 h post-transfection. Left: representative immunoblot image of the M–M co-IP experiment. Right: the normalized co-IP signal was plotted relative to the wild-type M protein for all mutants. Data represent the mean + standard deviation from four independent experiments and significant differences (*P ≤ 0.05) were assessed by Kruskal–Wallis test with Dunn’s correction for multiple comparisons

To find an explanation for the reduced M + E VLP production, M–E and M–M interactions were studied for all mutants and compared to the wild-type M. As reported before for IBV [45], cross-linking of M and E proteins was also required for MERS-CoV before detectable levels of M proteins co-precipitating with E became visible on western blot, indicating that M–E interactions might be weak and/or transient. Therefore, cells were treated with 0.8% formaldehyde before lysis and co-IP experiments were performed (Fig. 4A). In contrast to the M protein, the amount of E protein that co-precipitates with M remained too low to be well visualized even after cross-linking. This confirms what was seen for the VLPs, namely that only a very limited numbers of E proteins interacted with the wild-type M proteins.

M–E co-IP experiments performed with the three mutant M proteins (∆20_ct_, _211_DxE_213_A, and _199_KxGxYR_204_A) revealed that the _199_KxGxYR_204_A and ∆20_ct_ mutants showed a strongly reduced M–E interaction, whereas the _211_DxE_213_A mutant still interacted well with the E protein under these experimental conditions (Fig. 4A).

In contrast to M–E interactions, M–M interactions were strong enough to be visualized without previous cross-linking (Fig. 4B). These co-IP experiments showed that M–M interactions were normal for the ∆20_ct_ and _199_KxGxYR_204_A mutant. In contrast, the _211_DxE_213_A mutant showed a reduced M–M interacting capacity.

### Co-expression of the E protein can induce ER export of the ER-resident _211_DxE_213_A mutant M protein, but not of the ∆20_ct_ mutant

**Fig. 5:**
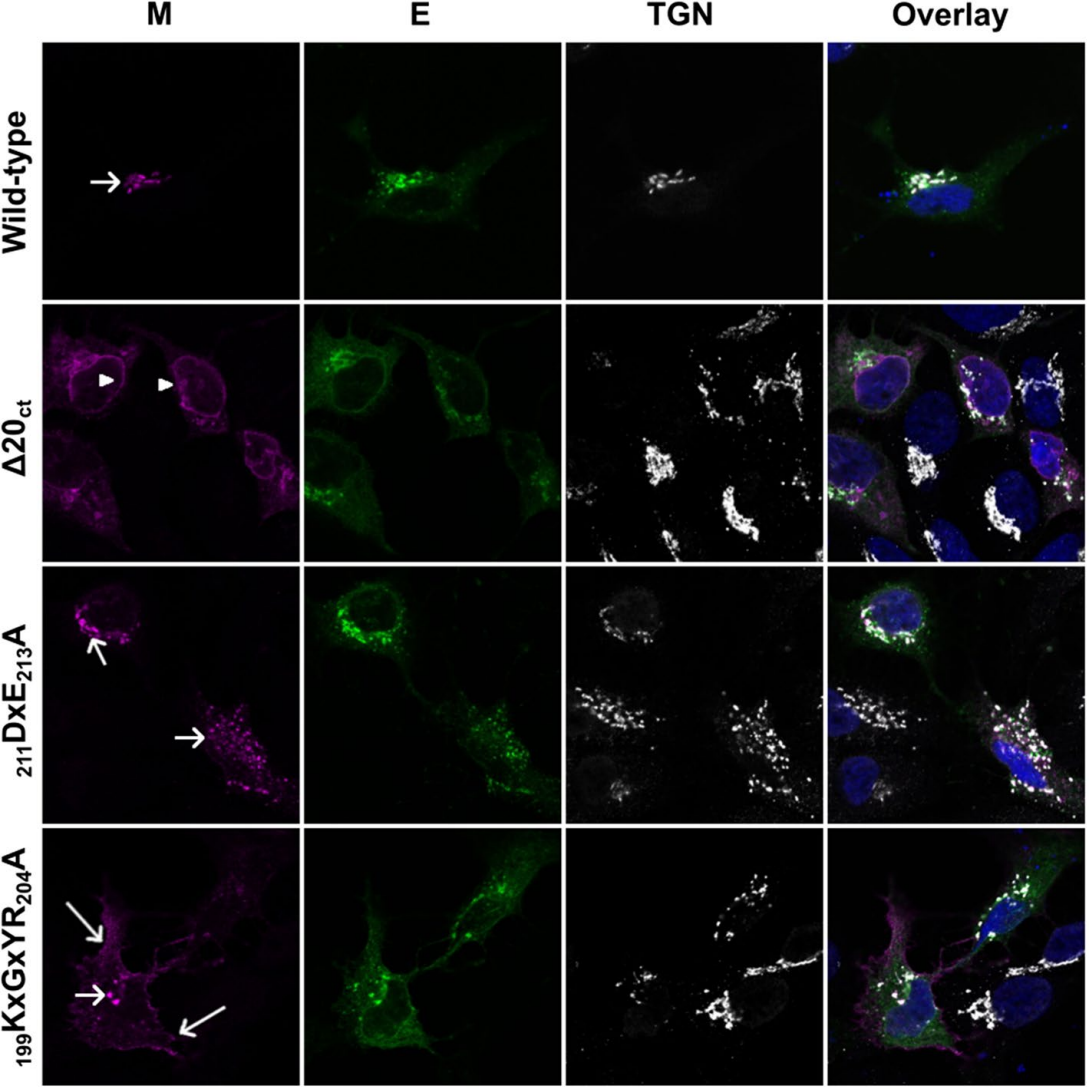
Subcellular localization of the wild-type M and ∆20_ct_, _211_DxE_213_A, and _199_KxGxYR_204_A mutants upon co-expression with the E-HSV protein. Huh-7 cells were transfected with equimolar amounts (125 ng of each/ 0.08 × 10^6^ cells) of an expression vector encoding the E-HSV protein and vectors encoding either the V5-tagged wild-type M, ∆20_ct_, _211_DxE_213_A, or _199_KxGxYR_204_A mutants. Sixteen hours post-transfection, cells were fixed and an immunofluorescence staining was performed against the V5-tag (magenta), the HSV-tag (green), and the TGN (white). Nuclei were visualized with DAPI (blue). Images were obtained with a confocal microscope (LSM 880, Zeiss). TGN, ER, and plasma membrane localizations are indicated with a short arrow, arrow head, and long arrow, respectively

To further validate the M–E co-IP results, we decided to test if the E protein could induce a switch in the subcellular localization of the (mutant) M proteins. Therefore, the subcellular localization of the wild-type and all mutant M proteins was assessed by immunofluorescence when expressed alone (Figure S4) or in combination with the E protein (Fig. 5). Co-expression of the E protein did not markedly change the localization of the wild-type protein, which still had a clear TGN localization upon M–E co-expression (Fig. 5). Interestingly, co-expression of E largely induced ER export of the ER-resident _211_DxE_213_A mutant M protein, but not of the ∆20_ct_ mutant. Co-expression of the E protein did not change the subcellular localization of the _199_KxGxYR_204_A mutant, which still showed a clear plasma membrane expression, similar to the single-expressed _199_KxGxYR_204_A mutant.

### Co-expression of the wild-type M protein can rescue the transport of all mutant proteins toward the TGN again

**Fig. 6:**
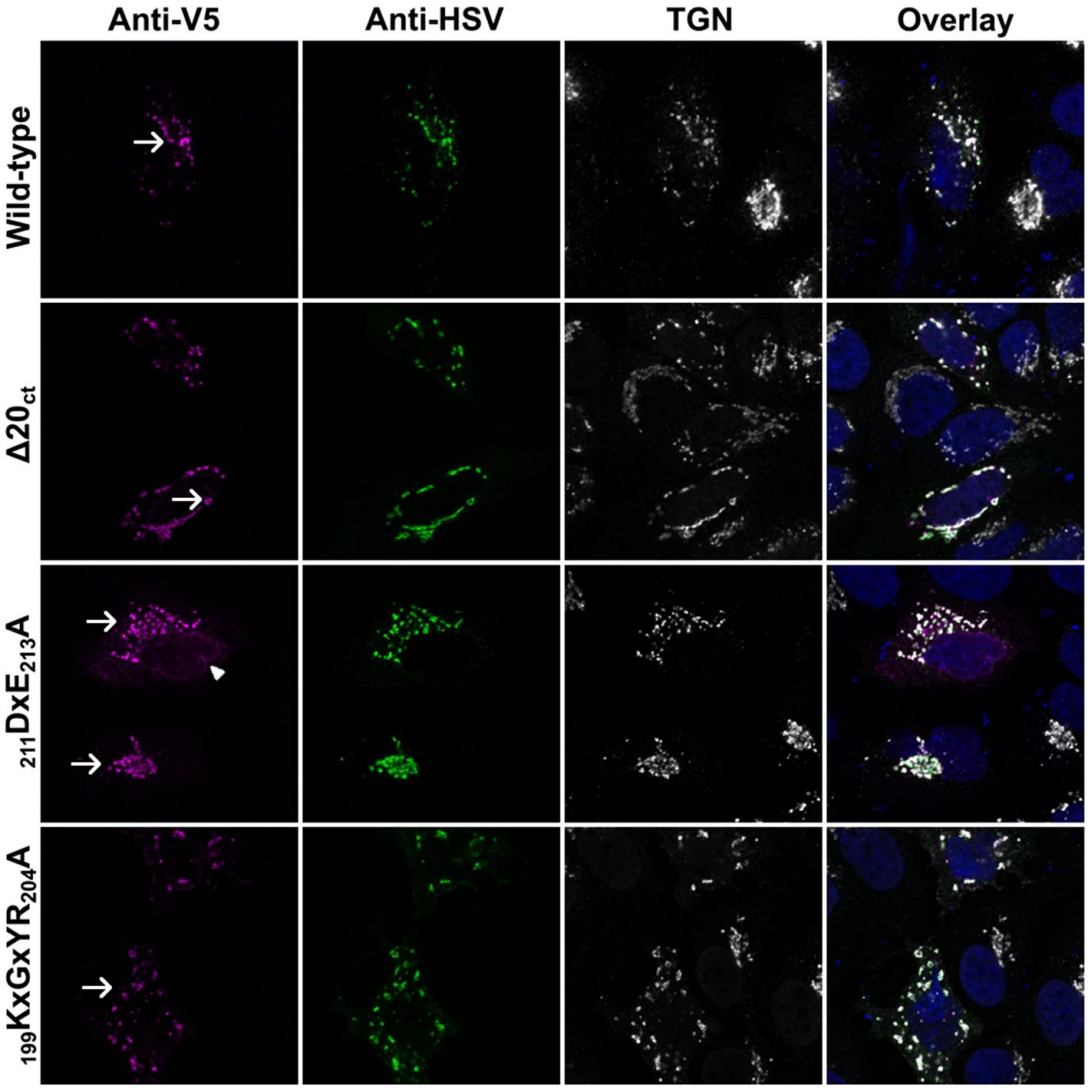
Subcellular localization of the wild-type M and ∆20_ct_, _211_DxE_213_A, and _199_KxGxYR_204_A mutants upon co-expression with the wild-type M-HSV protein. Huh-7 cells were transfected with equimolar amounts (125 ng of each/0.08 × 10^6^ cells) of an expression vector encoding the M-HSV-tagged protein and vectors encoding either the V5-tagged wild-type M, ∆20_ct_, _211_DxE_213_A, or _199_KxGxYR_204_A mutant. Sixteen hours post-transfection, cells were fixed and an immunofluorescence staining was performed against the V5-tag (magenta), the HSV-tag (green), and the TGN (white). Nuclei were visualized with DAPI (blue). Images were obtained with a confocal microscope (LSM 880, Zeiss). TGN and ER localizations are indicated with a short arrow and arrow head, respectively

To investigate if M–M interactions similarly affected the subcellular localization of the mutants, V5-tagged wild-type and mutant M proteins were co-expressed with an HSV-tagged wild-type M protein and the subcellular localization was visualized by confocal microscopy after immunofluorescence staining (Fig. 6). For all mutants, there was a clear signal in the TGN when the wild-type M protein was co-expressed. Only for the _211_DxE_213_A mutant, this transport rescue toward the TGN seemed to be incomplete, since there was still a remainder of the ER-like staining pattern visible in many cells.

### The _199_KxGxYR_204_A effect is mediated by its mislocalization

**Fig. 7:**
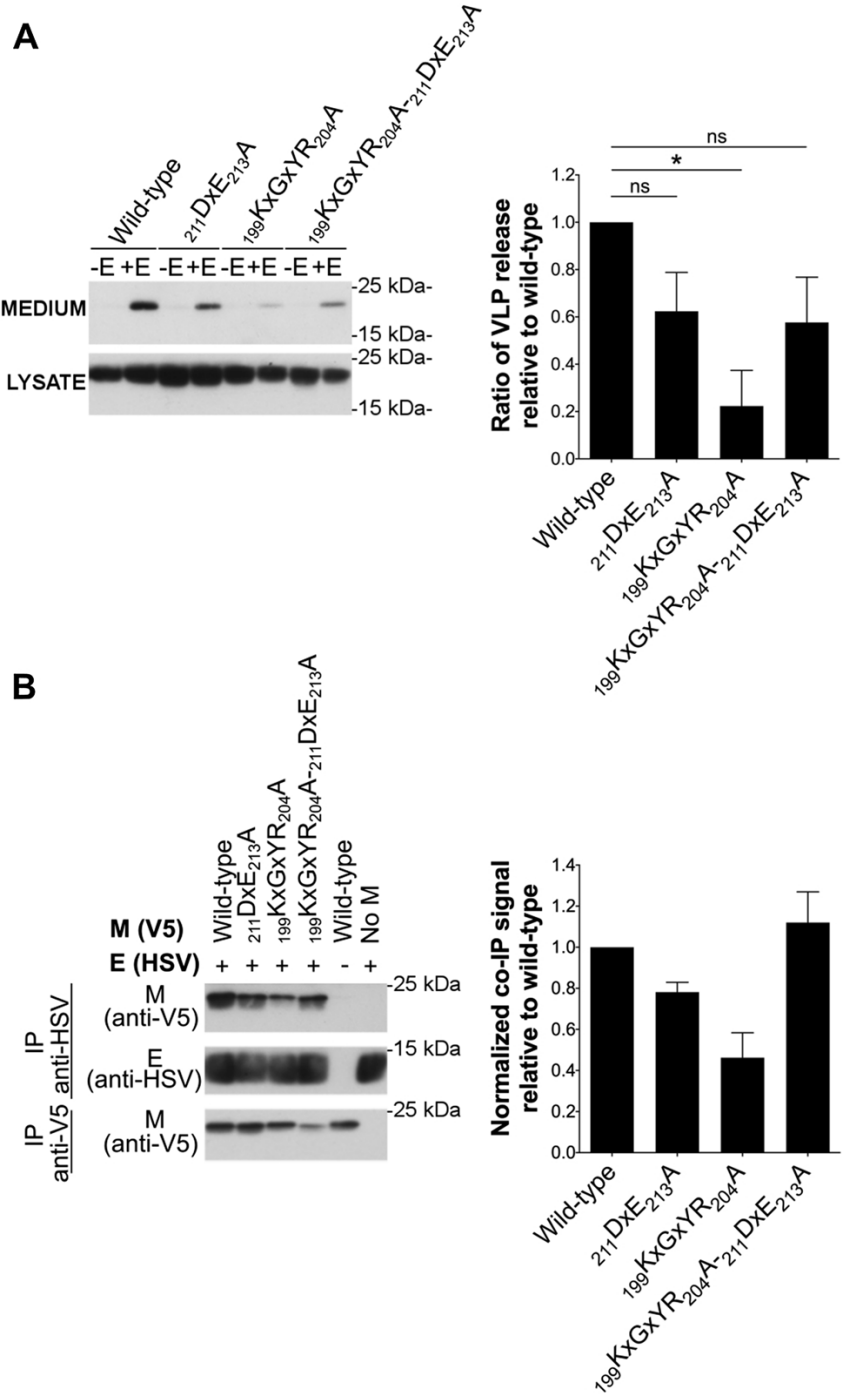
Assessment of the double _199_KxGxYR_204_A-_211_DxE_213_A mutant in VLP formation and M–E co-IP. **A** The VLP formation ability of the double _199_KxGxYR_204_A-_211_DxE_213_A mutant was assessed similarly as described above and compared to the single _211_DxE_213_A and _199_KxGxYR_204_A mutants. Data represent the mean + standard deviation from two (_211_DxE_213_A and _199_KxGxYR_204_A) or three (_199_KxGxYR_204_A-_211_DxE_213_A) independent experiments. Significant differences, as assessed by Kruskal–Wallis test with Dunn’s correction for multiple comparisons, are indicated with an asterisk (*P ≤ *0.05). **B** M–E interaction of the single and double mutants was assessed by co-IP as described above. Data represent the mean + standard deviation from three independent experiments

Since the _199_KxGxYR_204_ motif is present in the last 20 amino acids and since both the _199_KxGxYR_204_A and the ∆20_ct_ mutants had a clear effect on M–E interaction, it was further investigated whether the impaired M–E interaction seen with the _199_KxGxYR_204_A mutant could be explained by _199_KxGxYR_204_ being an M–E interacting motif or whether this was the result of the mislocalization of the _199_KxGxYR_204_A mutant protein. Therefore, an ER-localizing double mutant lacking both the _199_KxGxYR_204_ and the _211_DxE_213_ motif (_199_KxGxYR_204_A-_211_DxE_213_A) was constructed. We hypothesized that if the defect is due to the mislocalization induced by the _199_KxGxYR_204_A mutation, then addition of the _211_DxE_213_A mutation would rescue the M–E interaction and VLP formation. On the contrary, if the _199_KxGxYR_204_ motif is a direct M–E interacting motif, the M–E interaction and VLP formation capacity of the double mutant would be impaired. Figure 7 summarizes the results for the VLP formation (Fig. 7A) and M–E interaction (Fig. 7B) of the double _199_KxGxYR_204_A-_211_DxE_213_A mutant. These experiments showed that the _199_KxGxYR_204_A-_211_DxE_213_A double mutant behaved similar to the single _211_DxE_213_A mutant, and hence demonstrated that a normal M–E interaction can occur in absence of the _199_KxGxYR_204_ residues. This indicates that _199_KxGxYR_204_ is not a direct M–E interaction motif. Therefore, the reduced M–E interaction seen with the single _199_KxGxYR_204_A mutant is likely caused by its abolished intracellular retention. Moreover, this also means that lack of the _199_KxGxYR_204_ motif cannot account for the reduced M–E interaction seen with the ER-localizing ∆20_ct_ mutant (GxYR residues are part of the last 20 residues). This suggests that the last 20 residues of the M protein contain residues that are important for M–E interaction.

### A low E concentration-based VLP assay completely abolishes VLP formation for all mutants

**Fig. 8:**
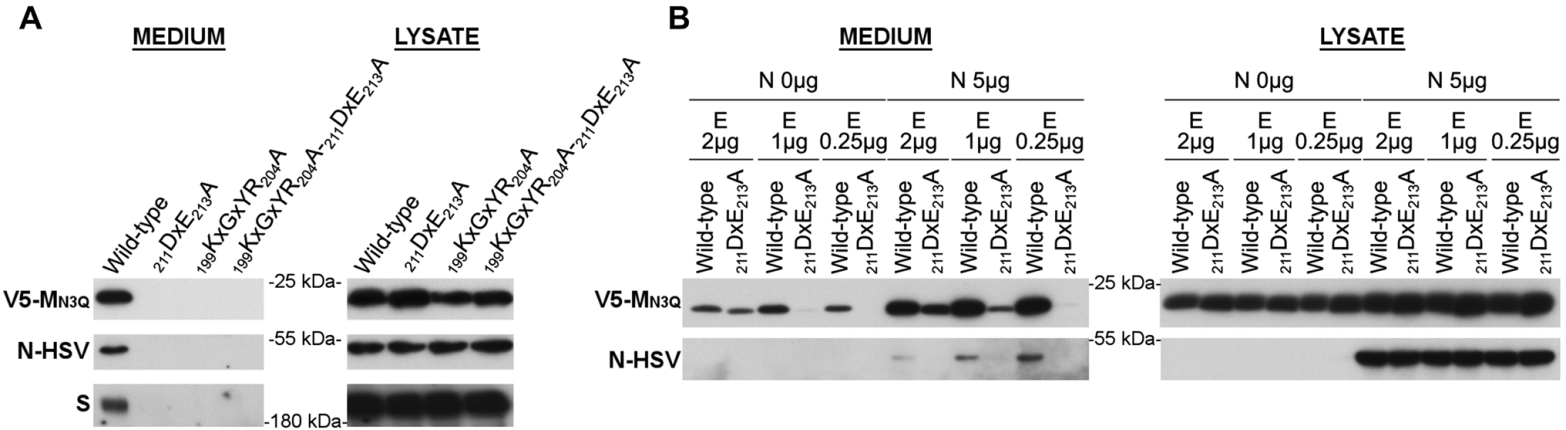
Effect of _211_DxE_213_A and _199_KxGxYR_204_A mutations on M + E_low_ + N VLP formation. **A** 2 × 10^6^ Huh-7-DPP4-KO cells were transfected with 2 µg of wild-type MERS-CoV V5-M_N3Q_, _211_DxE_213_A, _199_KxGxYR_204_A or _199_KxGxYR_204_A-_211_DxE_213_A-encoding plasmids in combination with MERS-CoV E-HSV-(0.25 µg), N-HSV-(5 µg) and S-encoding plasmids (3 µg). VLP secretion in the pelleted medium and expression of the M, N, and S proteins in the cell lysates were analyzed by western blot 48 h post-transfection. **B** 2 × 10^6^ Huh-7 cells were transfected with wild-type MERS-CoV V5-M_N3Q_ or _211_DxE_213_A-encoding plasmids in combination with various concentrations of E-HSV-encoding plasmids (2, 1, or 0.25 µg), in absence (0 µg) or presence (5 µg) of N-HSV-encoding plasmids. VLP formation and expression levels in the lysates were assessed 48 h post-transfection

To find out if the mutants would also impact the N incorporation into the VLPs, it was decided to additionally assess their effect in the M + E_low_ + N VLP assay. Surprisingly, however, in contrast to the basic M + E_high_ VLP assay where there was still at least a partial M + E assembly for the _211_DxE_213_A mutant M proteins, VLP signal was completely lost when using the M + E_low_ + N VLP assay (Fig. 8A). To find out whether this difference was caused by the N co-expression or by lowering the E concentration in this assay, wild-type and _211_DxE_213_A mutant M proteins were co-expressed with different concentrations of E-HSV (0.25, 1 or 2 µg), either in absence or presence (5 µg) of N proteins, and VLP formation was assessed (Fig. 8B). These experiments showed that both with and without N co-expression, DxE-VLPs could be formed in high E concentrations conditions, but not when using low E concentrations.

Conclusively, these data further indicate that two different MERS-CoV VLP assays can be obtained by changing the E concentration: (1) a basic M + E_high_(+ S) VLP assay which seems to be a good model to study the basic requirement for M–E interaction and assembly but for which assembly results in the formation of nucleocapsid-empty particles and might partially take place in the RER (cfr assembly with _211_DxE_213_A mutant); and (2) a low E concentration-based VLP assay that allows for N incorporation (M + E_low_ + N(+ S)), which might represent the fully assembled, but viral RNA-free, virus particle. Moreover, these data suggest that intracellular trafficking and retention of the M protein plays a major role in the generation of fully assembled particles.

### Both _211_DxE_213_A and _199_KxGxYR_204_A mutations in an infectious MERS-CoV cDNA clone completely abolish the infectious virus production

**Fig. 9:**
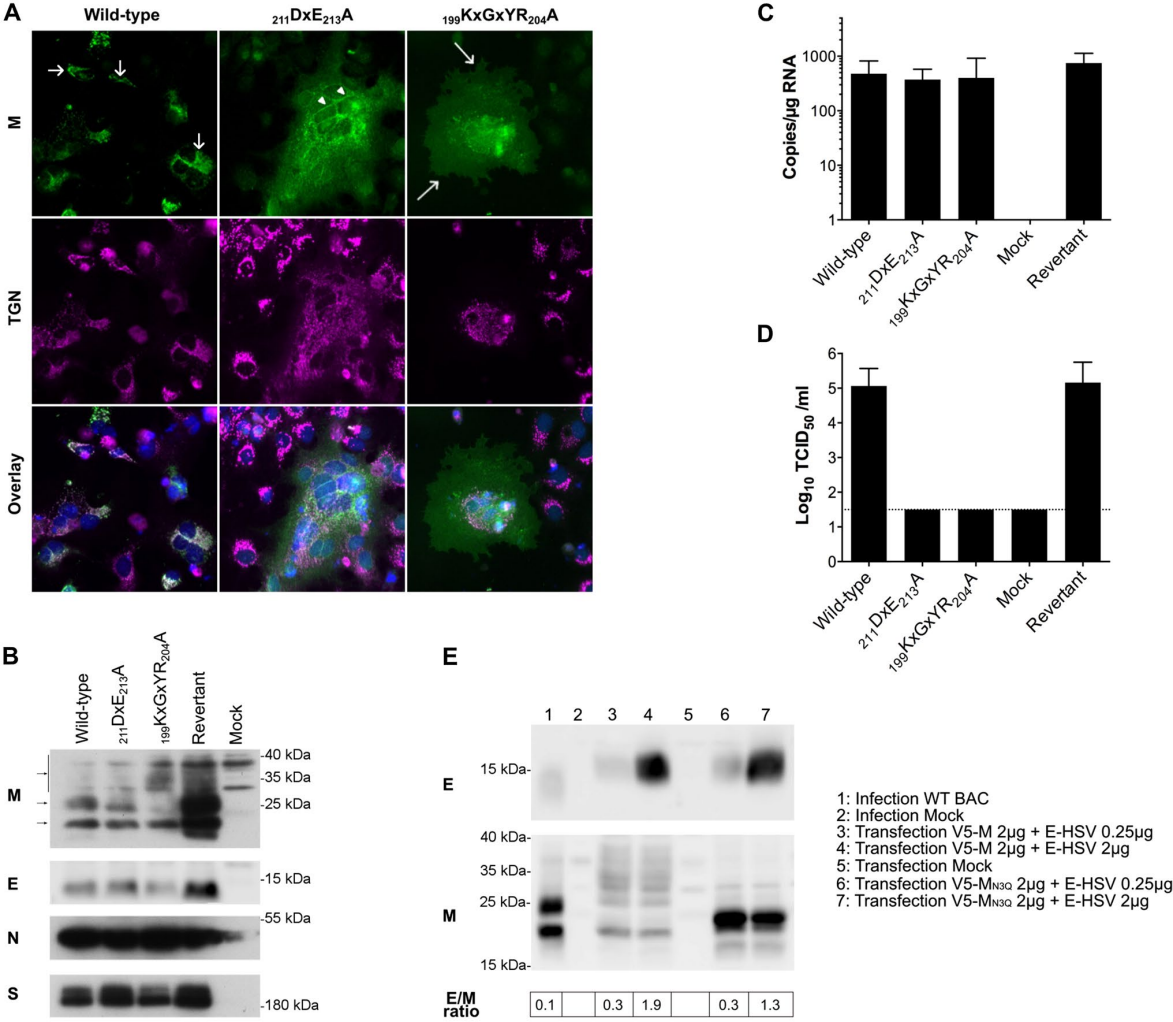
Protein expression and infectious virus production in Huh-7 cells transfected with wild-type and mutant MERS-CoV cDNA constructs. **A** Intracellular M protein expression was assessed by immunofluorescence staining for wild-type and _211_DxE_213_A or _199_KxGxYR_204_A mutant cDNA constructs. Therefore, cells were fixed 4 days post-transfection and an immunofluorescence staining was performed against the M protein (green) and the TGN (magenta). Nuclei were visualized with DAPI (blue). Images were obtained with an EVOS M5000 imaging system. TGN, ER, and plasma membrane localizations are indicated with a short arrow, arrow head, and long arrow, respectively. **B** M, E, N, and S expression levels were assessed by western blot for all cDNA constructs on cell lysates collected 2 days post-transfection. **C** Wild-type or mutant MERS-CoV cDNA clones were used to transfect Huh-7 cells, and 24 h post-transfection, RNA was extracted from duplicate wells and analyzed by qRT-PCR to determine the replication capacity for all constructs. **D** Four days post-transfection, the supernatant was collected from duplicate wells for all constructs and release of infectious virus was determined by infectivity titrations in Huh-7 cells. The dashed line indicates the detection limit. Data in C and D represent the mean + standard deviation from three independent experiments. **E** Immunoblot images showing E and M protein expression levels in wild-type BAC-transfected and pcDNA-transfected Huh-7 cells 2 days post-transfection. The E/M ratio was quantified by calculating E and M signals on the western blots using the Image J software and its band quantification function

To further confirm the importance of the M trafficking for infectious virus assembly, the _211_DxE_213_ and _199_KxGxYR_204_ motifs were mutated into alanine in an infectious MERS-CoV cDNA clone [44]. In agreement with what was seen with the single-expressed M proteins, the subcellular localization of M was clearly changed for the mutant constructs, i.e., an ER-like staining pattern for the _211_DxE_213_A mutant and leakage to the plasma membrane for the _199_KxGxYR_204_A mutant (Fig. 9A). As assessed by qRT-PCR on cell lysates 24 hpt, all constructs (wild-type, _211_DxE_213_A, _199_KxGxYR_204_A, and a revertant wild-type mutant made starting from the _199_KxGxYR_204_A-cDNA) replicated equally well upon transfection in Huh-7 cells (Fig. 9C). However, despite RNA replication and expression of the structural proteins similar to the wild-type virus (Fig. 9B), there was no detectable production of infectious virus when either the _211_DxE_213_ or the _199_KxGxYR_204_ motif were mutated (Fig. 9D). Altogether, these data confirm that the _211_DxE_213_-mediated ER export and the _199_KxGxYR_204_-mediated intracellular retention of the MERS-CoV M protein are both required for infectious particle assembly.

As low E concentrations seemed to be the key to produce natural N-containing VLPs, questions arose on the E concentrations during natural infections. Therefore, E and M proteins expression levels in wild-type BAC launched infected cells were assessed and compared to the transfection conditions used in the E_low_ and E_high_ VLP assays (Fig. 9E). Under similar E expression conditions, M expression levels were even higher during infection than in the M + E_low_ transfection conditions (compare lane 1 with lane 3/6). To compare the difference of protein expression between our different conditions, we calculated a ratio that is based on the quantification of the M and E protein signal on the western blots. The M + E_high_ transfection conditions showed an inverted E/M ratio compared to the infected cells (compare lane 1 with lane 4/7). Taken together, these results show that during infection, a low E/M ratio is indeed present and even lower than in the low E-based VLP system.

## Discussion

The CoV assembly process is spatiotemporally regulated and the efficiency of this process is determined by specific signals in the viral proteins for trafficking to, and protein–protein interactions at, the assembly site (i.e., the ERGIC/cis-Golgi for most CoVs) [12, 13]. Assembly can only correctly occur if all virion-associated components are directed to the assembly site and if they interact with each other, but this complex process still remains largely elusive. For CoVs, the M protein seems to be the driving force during assembly by interactions with the other structural proteins [16–18]. Previously, we identified two motifs that are important for the intracellular trafficking (_211_DxE_213_) and retention (_199_KxGxYR_204_) of the single-expressed MERS-CoV M protein [27]. The present study shows that the intracellular trafficking and localization of the M protein determined by both signals greatly impact its capacity to mediate viral assembly, which was assessed by performing VLP assays with mutant M proteins and by introducing these mutations in an infectious MERS-CoV cDNA clone.

To perform the VLPs assays, Huh-7 cells were chosen in the present study, since these cells gave a stable and uniform expression of all proteins in all co-expression conditions (in contrast to HEK293T cells), and since these cells were used for the studies with the infectious MERS-CoV cDNA as well. Co-transfection of Huh-7 cells with minimal concentrations of M- (2 µg/2 × 10^6^ cells) and E-encoding plasmids (0.5 µg/2 × 10^6^ cells) was required, but sufficient, to induce MERS-CoV VLP formation in these cells. Many other similarities were found with previous reports on other CoVs [6, 18], including the fact that N-glycans on the M protein did not play a role in the formation of VLPs, and that S co-expression did not affect the VLP formation but S proteins were incorporated in the VLPs when co-expressed with M and E. Here, these MERS-CoV M + E + S VLPs were only visible upon knockout of the MERS-CoV receptor, DPP4, in the Huh-7 cells. In addition, both M and E proteins also showed a restricted secretion when expressed alone, as for other CoVs [7, 45–47]. It remains to be investigated whether these individually expressed proteins were capable of forming VLPs on their own or whether secretion occurred in other non-VLP-specific structures, as shown for SARS-CoV-2 M [48]. At least for the M protein, it was shown here that this observation might notably depend on the used experimental conditions, since the ‘M-only’ secretion artificially increased when using a C-terminally V5-tagged M protein.

It has been described that the additional co-expression of the nucleocapsid (N) protein can induce more efficient release of CoV VLPs, and in some studies, N co-expression was even crucial to detect the VLP formation [29, 31, 39]. Here, it was shown that also for MERS-CoV, N-co-expression allowed to detect and to saturate the VLP signal in otherwise suboptimal, lower E concentration conditions (0.25 µg/2 × 10^6^ cells). Remarkably, in higher E concentration conditions, N incorporation was lost. So far, it is not clear why nucleocapsid-empty particles tended to be formed in high E concentration conditions. However, some peculiar findings with the ER-retained M protein mutant, _211_DxE_213_A, might hint toward an M–E interaction and M + E assembly at the RER in high E concentration conditions, which seems to be absent in low E concentration conditions. Indeed, when the E protein was co-expressed in high, equimolar concentrations as the M protein, the _211_DxE_213_A mutant showed a normal M–E interaction, and E protein co-expression even helped the _211_DxE_213_A mutant to be exported from the ER. Moreover, M + E VLP assembly with the _211_DxE_213_A mutant was still noticeable in high E concentrations conditions but was completely lost in low E concentrations conditions. Together with the observation that nucleocapsid-empty particles were formed with the wild-type M protein in high E concentrations conditions, this raises the hypothesis that high E concentrations might induce ‘too early’ particle formation in the RER upon translation of M and E proteins, i.e., before M (or E) proteins had the opportunity to interact with the N proteins in the cytoplasm. However, more in-depth imaging of the exact VLP assembly sites of both the M + E_high_ and M + E_low_ + N VLPs will be required to clarify this hypothesis. Nonetheless, ER export of M was not induced upon mutation of its _211_DxE_213_ motif in the infectious MERS-CoV cDNA clone, indicating that this ER-located M–E interaction might be an artificial observation caused by supra-physiological concentrations of the E protein in our E_high_ transfection conditions. Indeed, comparative immunoblots confirmed that low E concentrations used in the E_low_ transfection conditions better reflect the low E/M ratio present during MERS-CoV infection in Huh-7 cells.

Introduction of the _211_DxE_213_A or _199_KxGxYR_204_A mutations in an infectious MERS-CoV cDNA further confirmed that the low E concentration-based VLP assay was the most physiologically relevant assay, as the latter also showed a complete loss of virus assembly with all the mutant M proteins. Nonetheless, we believe that the M + E_high_ VLP assay might be a great asset too, especially if basic M–E interactions or assembly need to be assessed in absence of other structural proteins. In addition, blocking the M protein in the ER by _211_DxE_213_A mutation and co-expressing equimolar amounts of E protein seems to be a very elegant system to co-localize M and E proteins and to identify or rule-out certain motifs as being involved in M–E interaction and basic particle formation, as proven by the double _199_KxGxYR_204_A-_211_DxE_213_A mutant in this study. Using the M + E_high_ VLP assay, it was also noticed that the other ER-retained mutant, ∆20_ct_, severely reduced basic M + E VLP formation, caused by a reduced M–E interaction. The impaired M–E interaction for the ∆20_ct_ mutant was further supported by immunofluorescent staining, showing that, in contrast to the _211_DxE_213_A mutant, co-expression of E could not help in ER export of the ER-resident ∆20_ct_ mutant protein. So, it seems that the M–E interaction and E-induced ER export of the M protein requires a full-length M protein, or at least the presence of non-_211_DxE_213_-related residues in the last 20 amino acids of the M protein. Moreover, the fact that the double _199_KxGxYR_204_A-_211_DxE_213_A mutant showed a normal M–E interaction indicates that the reduced M–E interaction seen with the ∆20_ct_ mutant cannot be explained by the absence of the _199_KxGxYR_204_ motif (GxYR are part of the last 20 amino acids), and hence that other residues within these last 20 amino acids might play a role in the M + E interaction and basic M + E VLP assembly. Based on previous reports on MHV, another *Betacoronavirus*, the utmost two C-terminal residues on the M protein are critical for virus assembly, presumably by disturbing M–E interaction, since M + E VLP production was reported to be completely abrogated if the utmost two C-terminal amino acids were deleted [18]. In contrast to MHV, deleting the last 2 amino acids of the MERS-CoV M protein had only a slight reducing effect on basic M + E_high_ VLP formation (data not shown), so more mutational analyses will be required to investigated which residues in the last 20 amino acids are required for correct M–E interaction.

Up to date, it remains elusive how the _199_KxGxYR_204_ motif succeeds in retaining the M protein intracellularly, and hence why its mutation results in increased plasma membrane expression of the M protein [27]. Here it was shown that the _199_KxGxYR_204_A mutant showed a decreased M–E interaction and had a major impact on the basic M + E_high_ VLP production. Moreover, using the double _199_KxGxYR_204_A-_211_DxE_213_A mutant, it was excluded that _199_KxGxYR_204_ is a direct M–E interaction motif, indicating that the reduced M–E interaction solely resulted from the M mislocalization induced upon mutation of this motif. With regard to the hypothesis that M–E interactions and M + E assembly might already start in the RER in our basic M + E_high_ VLP conditions as stated above, questions arise whether mutation of the _199_KxGxYR_204_ motif might not (additionally) speed up ER exit or prevent ER retrieval. This would also explain the lack of the EndoH-sensitive, high-mannose form of the M protein [27] in the _199_KxGxYR_204_ mutated infectious cDNA clone (Fig. 9B, middle arrow).

M–M interactions were normal for the _199_KxGxYR_204_A mutant, confirming previous findings that _199_KxGxYR_204_ is not an oligomerization motif to keep the M protein in the TGN [27], and indicating that M–M interactions do not necessarily take place upon Golgi/TGN retention for MERS-CoV, as suggested before for MHV [49]. From the present study, it is hard to assess where those M–M interactions are initiated, since the two ER-localizing M proteins (_211_DxE_213_A and ∆20_ct_) showed a different M–M interacting capacity. So far, it is not clear why the _211_DxE_213_A mutant showed a decreased M–M interaction, whereas the ∆20_ct_ mutant did not. However, although both have a defect in ER export, these two mutants present some other differences in intracellular retention/trafficking. The deletion of the last 20 residues induces a strong retention of the protein in the ER because of the lack of the DxE signal but on the other hand, proteins that do leave the ER are no longer retained in the TGN because of the additional lack of the _199_KxGxYR_204_ motif. Therefore, compared to the _211_DxE_213_A mutant, the ∆20_ct_ mutant has a higher cell surface expression (Figure S4C). This difference of repartition of the protein along the secretory pathway may be responsible for the difference in M–M interaction. M proteins of betacoronaviruses form dimers and can also be found in higher order oligomerization states [50]. Non-reducing gel analyses with the _211_DxE_213_A mutant showed that the dimerization does not seem to be affected for this mutant (Figure S5), raising the possibility that the difference in M–M interaction lies in the higher order oligomers. Since more ∆20_ct_ M proteins traffic to the plasma membrane than the _211_DxE_213_A mutant, it is possible that higher state oligomer formation occurs in a post-ER compartment, and hence make up the difference between both mutants, but more in-depth analyses of these oligomers will be required.

With the outbreak of the novel human SARS-CoV-2, the need for effective antivirals against CoVs had become urgent. Given this urgency, many studies have focused on repurposing the use of yet approved drugs designed to speed up the antiviral development process at the beginning of the epidemic. For the future, however, it might be interesting to further elucidate coronavirus-specific targets. The _199_KxGxYR_204_ motif within the C-tail of the M protein might be such an interesting target. Apart from some small variations, such as RxGxYK (SARS-CoVs) or KxGxYS (Alphacoronaviruses), this motif is quite well conserved among many CoVs, so it warrants further investigation if this motif is also necessary for the correct M localization and/or for the assembly of other coronaviruses. For MHV, mutation of only the tyrosine residue within this motif did slightly impact its localization but did not severely impact the M + E VLP formation [18], so it would be interesting to see if mutation of the full motif would change this. Nonetheless, the present study shows that targeting the function of the _199_KxGxYR_204_ motif reduces at least MERS-CoV spread by disturbing the assembly process. In addition, one might expect that interference with its function can additionally make the infected cell more visible for the immune system by increasing the plasma membrane expression of the M protein [27], thereby helping the host to clear the infection more rapidly. Given its conservation and its crucial role in virus assembly, subsequent studies should focus on how this motif succeeds in retaining the M protein intracellularly and if therapeutic interference with this process is possible.

### Supplementary Information

Below is the link to the electronic supplementary material.

Supplementary file1 (DOCX 15944 kb)

## Data Availability

The raw data that support the findings of this study are available from the corresponding author upon request.
