# Supplementary material for: The KxGxYR and DxE motifs in the C-tail of the Middle East respiratory syndrome coronavirus membrane protein are crucial for infectious virus assembly
Source: Cell Mol Life Sci. 2023 Nov 9;80(12):353. doi: 10.1007/s00018-023-05008-y (PMC10632273; doi:10.1007/s00018-023-05008-y)
Supplement: Supplementary file 1 — Supplementary file1 (DOCX 15944 kb) [file 18_2023_5008_MOESM1_ESM.docx]

**The KxGxYR and DxE motifs in the C-tail of the Middle East respiratory syndrome coronavirus membrane protein are crucial for infectious virus assembly**

Lowiese Desmarets^1^, Adeline Danneels^1^, Julien Burlaud-Gaillard^2,3^, Emmanuelle Blanchard^2,3^, Jean Dubuisson^1^, Sandrine Belouzard^1*^

^1^ Université de Lille, CNRS, Inserm, CHU Lille, Institut Pasteur de Lille, U1019 - UMR 9017 - CIIL- Center for Infection and Immunity of Lille, F-59000 Lille, France.

^2^ INSERM U1259 MAVIVH, Université de Tours and CHRU de Tours, Tours, France.

^3^ Plate-Forme IBiSA de Microscopie Electronique, Université de Tours and CHRU de Tours, Tours, France.

^*^ Corresponding author: sandrine.belouzard@ibl.cnrs.fr

**SUPPLEMENTARY INFORMATION**

**S1. Optimization of the VLP assay**

**S1.1 HEK293T cells are not suitable to assess the formation of MERS-CoV VLPs.**


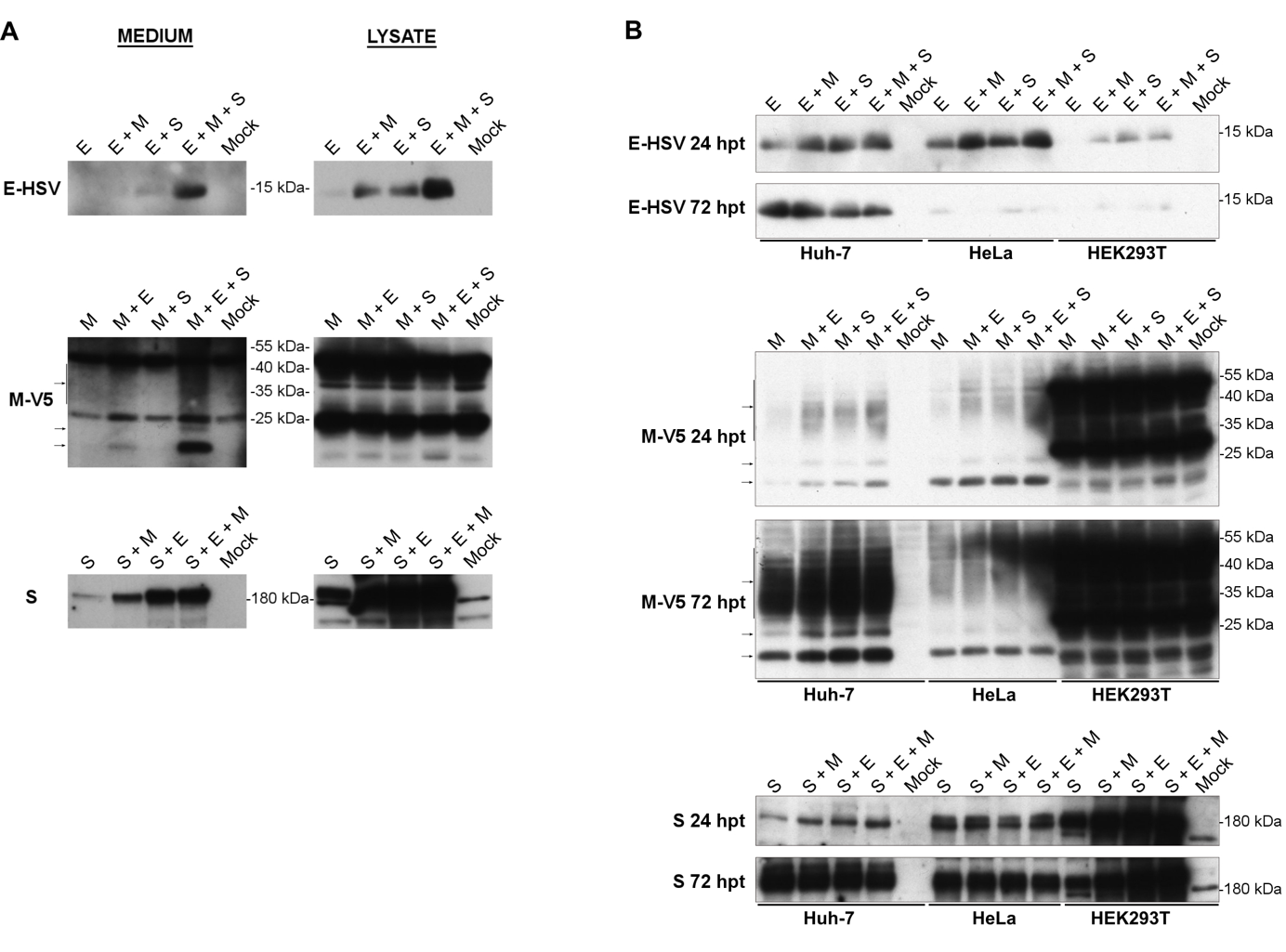
First, the production of VLPs in HEK293T cells was tested. 2x10^6^ HEK293T cells were transfected with MERS-CoV M-V5 (2µg), E-HSV (1µg) and S (3µg)-encoding plasmids, either alone or in combination. Unfortunately, the intracellular expression levels of the proteins varied greatly in the different conditions (single vs multi-transfection), making it impossible to assess whether secreted protein levels were increased by VLP formation or rather increased by non-specific secretion of the single proteins (Figure S1A). Moreover, M visualization was seriously hampered by non-specific bands on blots in the HEK293T cell lysates and E protein expression was not stable over time (Figure S1B). In Huh-7 cells on the contrary, a stable and uniform expression level was noticed for all proteins in all conditions over time in our hands and hence these cells were used to further assess the MERS-CoV VLP formation.

**Figure S1. MERS-CoV VLP formation and protein expression levels in HEK293T cells. A.** Representative immunoblot images showing the MERS-CoV E, M and S proteins in the pelleted supernatant (= medium) and the expression levels of the proteins in the cells (= lysate) 48 h after single or combined transfection of 1 µg of E-HSV-, 2 µg of glycosylated M-V5-, and 3 µg of S-encoding plasmids in 2x10^6^ Hek293T cells. Empty vector was used to complete the total amount of transfected DNA to 6 µg if necessary. Mock refers to the transfection of an equal amount of empty vector. **B.** Representative immunoblot images showing the expression levels of the MERS-CoV E, M and S proteins 24 h and 72 h after single or combined transfection of 1 µg of E-HSV-, 2 µg of glycosylated M-V5, and 3 µg of S-encoding plasmids in various cell lines (Huh-7, HeLa, and HEK293T cells). Empty vector was used to complete the total amount of transfected DNA to 6 µg if necessary. Mock refers to empty vector transfection.

**S1.2 Optimization of the VLP assay in Huh-7 cells.**


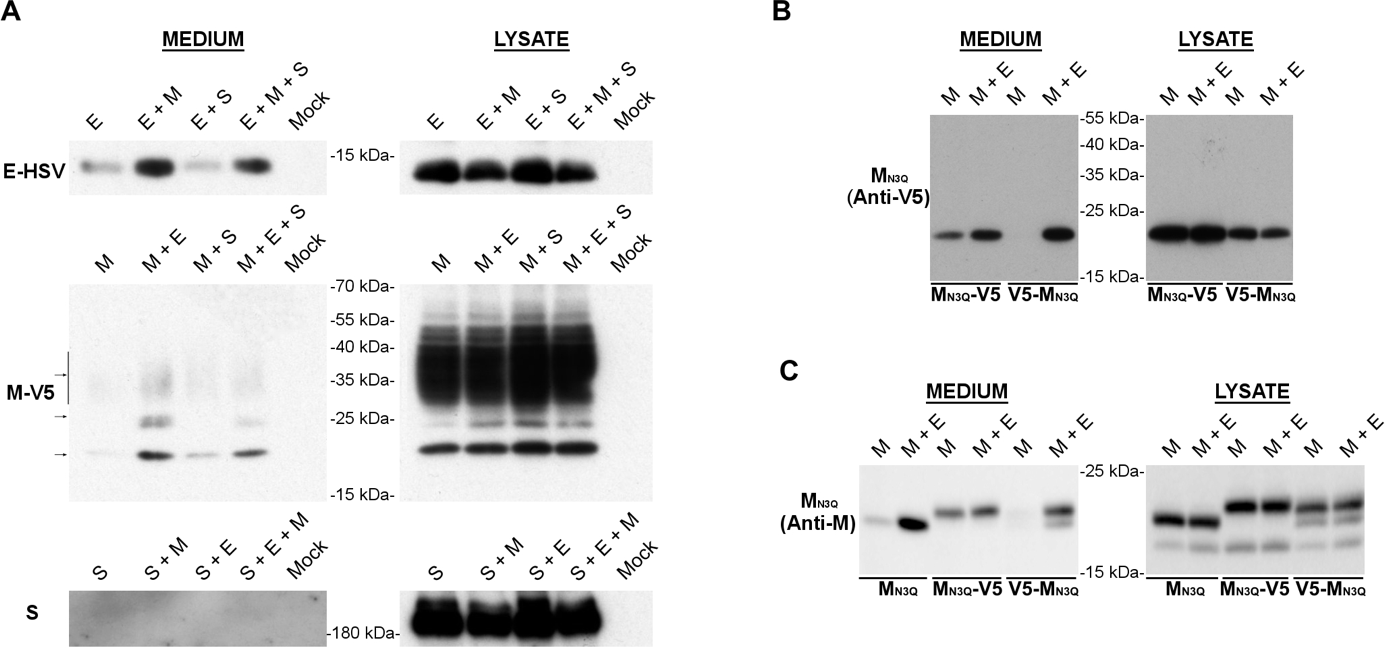
Figure S2A shows the western blot detection of the E-HSV, M-V5 and S proteins in the pelleted supernatant (= medium) and the expression levels of the proteins in Huh-7 cells (= lysate) upon single- and multiple protein expression. In contrast to M and E, none of the tested conditions showed an S signal in the pelleted medium for Huh-7 cells (Figure S2A). We reasoned that the lack of visible S incorporation in M+E VLPs was due to the reattachment of the produced M+E+S VLPs to the cell surface, since Huh-7 cells express the DPP4 receptor that is recognized by the MERS-CoV S protein. Secretion of the E protein was visible in all conditions, but this secretion was enhanced in the conditions where M was present. Similar results were obtained for the C-terminally tagged M-V5 protein, namely a clear secretion when expressed alone, but an enhancement in conditions where E was present. There was no further enhancement upon co-expression of S. A comparable M secretion pattern was still visible when using a N-glycosylation site mutant (N3Q) (Figure S2B). Interestingly, the ‘M-only’ secretion seemed to become much weaker when using an N-terminally instead of a C-terminally V5-tagged M protein (Figure S2B). Comparison with the untagged M revealed that the larger ‘M-only’ secretion seen with the C-terminally M protein was indeed artificially induced by the tag position (Figure S2C). These results show that N-glycans on the M protein are not involved in the VLP formation, and that the position of the tag can affect the release of the single-expressed M protein.

**Figure S2. Effect of M glycans and tag position on the MERS-CoV VLP formation in Huh-7 cells. A.** Representative immunoblot images showing the MERS-CoV E, M and S proteins in the pelleted supernatant (= medium) and the expression levels of the proteins in the cells (= lysate) 48 h after single or combined transfection of 1 µg of E-HSV-, 2 µg of glycosylated M-V5-, and 3 µg of S-encoding plasmids in 2x10^6^ Huh-7 cells. Mock refers to the transfection of an equal amount of empty vector. M blots were revealed using an anti-V5 antibody. **B**. Representative immunoblot images showing the extracellular release (= medium) and expression (= lysate) of unglycosylated M_N3Q_-V5 and V5-M_N3Q_ proteins upon single expression or combined with the E-HSV protein in Huh-7 cells. Blots were revealed using an anti-V5 antibody. **C.** Representative immunoblot images showing the extracellular release (= medium) and expression (= lysate) of unglycosylated untagged M_N3Q_ or tagged M_N3Q_-V5 and V5-M_N3Q_ proteins upon single expression or combined with the E-HSV protein in Huh-7 cells. Blots were revealed using an anti-MERS-CoV-M antibody.

**S1.3 Minimal amounts of M and E are required for the M+E VLP formation.**

Structural proteins are not present in equal amounts during CoV infection. To assess if changes in concentrations would impact M+E VLP formation, 2x10^6^ Huh-7 cells were co-transfected with 1 or 2 µg V5-M_N3Q_ -encoding plasmid and various concentrations of E-HSV-encoding plasmid, ranging from 0.25 to 2 µg. VLPs were only detectable at higher M concentrations (2 µg/2x10^6^ cells), whereas lowering the E concentration down to 0.5 µg was sufficient to clearly see the VLP-associated M secretion (Figure S3A). The E protein was barely detectable in the lysate and VLP fraction at this concentration and required transfection with at least 1 µg of E-encoding plasmid to be visualized (Figure S3B). At lower E concentrations (0.25 µg), the M+E VLP formation became inefficient (Figure S3A). Increasing the E concentration augmented the release of the E protein in both single and M+E expression conditions, but it did not further increase the release of the M protein, indicating a saturation of the VLP formation from 0.5 µg of E-encoding plasmids onwards (Figure S3A and B). In higher concentrations (2 and 4 µg), co-expression of E seemed to even slightly suppress M expression levels in the lysate, but this had no effect on the VLP secretion levels. All together, these data show that both M and E proteins are crucial for MERS-CoV VLP formation, but these M+E VLPs seem to require a minimal expression level of both M and E proteins for their assembly.


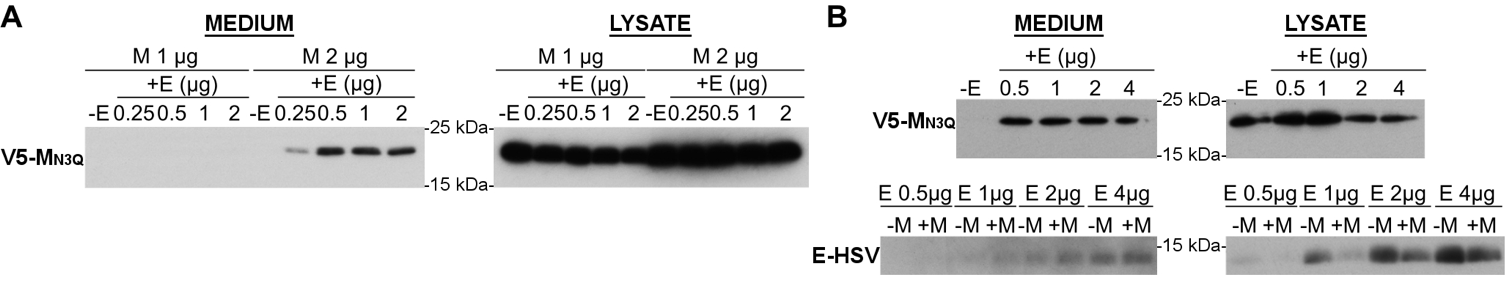


**Figure S3. Minimal amount of M and E proteins required for M+E VLP detection.** **A.** Representative immunoblot images showing the extracellular release (= medium) and expression levels (= lysate) of V5-M_N3Q_ proteins upon co-transfection of 1 or 2 µg V5-M_N3Q_-encoding vector with various concentrations of E-HSV-encoding vector (0, 0.25, 0.5, 1 or 2 µg) in 2x10^6^ Huh-7 cells. **B.** Extracellular release and expression levels of both V5-M_N3Q_ and E-HSV proteins upon co-transfection of 2x10^6^ Huh-7 cells with 2 µg V5-M_N3Q_-encoding vector and various concentrations of MERS-CoV E-HSV-encoding vector (0, 0.5, 1, 2, and 4 µg).

**S2. Other supplementary figures**


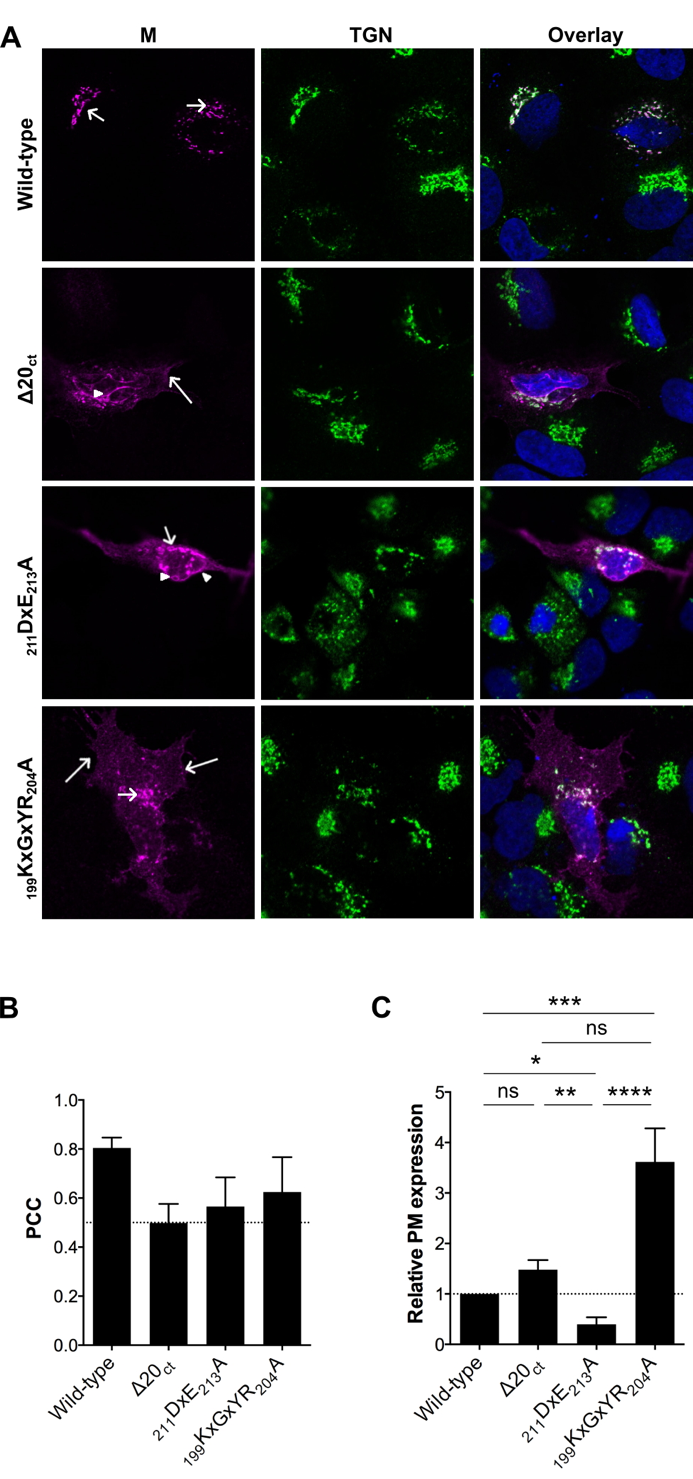


**Figure S4. Subcellular localization of the wild-type M, ∆20_ct_, _211_DxE_213_A, and _199_KxGxYR_204_A mutants when expressed alone. A.** Huh-7 cells were transfected with expression vectors encoding for V5-tagged wild-type M, ∆20_ct_, _211_DxE_213_A, and _199_KxGxYR_204_A mutants. Sixteen hours post-transfection, cells were fixed and an immunofluorescence staining was performed against the V5 tag (magenta) and TGN46 (green). Nuclei were visualized with DAPI (blue). Coverslips were mounted and the subcellular localization was analyzed by confocal microscopy (LSM 880, Zeiss). TGN, ER and plasma membrane localizations are indicated with a short arrow, arrow-head and long arrow, respectively. **B.** The Pearson’s correlation coefficients were calculated to quantify the extent of TGN localization in Huh-7 cells for each mutant. **C.** Huh-7 cells were transfected with vectors encoding for HiBit-M_N3Q_, HiBit-M_N3Q_-∆20_ct,_ HiBit-M_N3Q_-_211_DxE_213_A and HiBit-M_N3Q_-_199_KxGxYR_204_A. 16h post-transfection, the extent of plasma membrane expression of the mutants was quantified by means of the Nano-Glo^®^ HiBit Extracellular Detection System, and total protein expression was quantified for all constructs using the Nano-Glo^®^ HiBit Lytic Detection System. The ratio plasma membrane signal/total signal was calculated for all constructs and expressed relative to the wild-type protein. Significant differences, as assessed by the Kruskal-Wallis test with Dunn’s correction for multiple comparisons, are indicated with an asterisk (P ≤ 0.05).

**
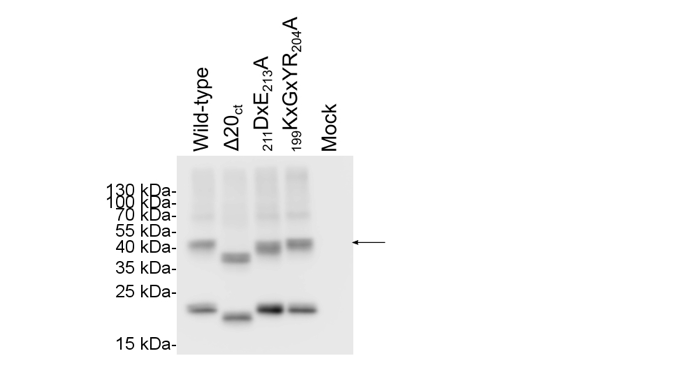
**

**Figure S5. M protein dimers.** Representative immunoblot image showing monomeric and dimeric (arrow) M proteins after transfection of Huh-7 cells with V5-M_N3Q_, V5-M_N3Q_-∆20_ct_, V5-M_N3Q_-_211_DxE_213_A or V5-M_N3Q_-_199_KxGxYR_204_A-encoding plasmids.
